# The association of prenatal ambient air pollution with placental epigenetic gestational age at birth

**DOI:** 10.1097/EE9.0000000000000384

**Published:** 2025-05-05

**Authors:** Zhengting He, Ashley Y. Song, Rose Schrott, Jason I. Feinberg, Kelly M. Bakulski, Kelly S. Benke, Lisa A. Croen, Irva Hertz-Picciotto, Rebecca J. Schmidt, Kristen Lyall, Craig J. Newschaffer, M. Daniele Fallin, Heather E. Volk, Christine Ladd-Acosta

**Affiliations:** a Department of Epidemiology, Bloomberg School of Public Health, Johns Hopkins University, Baltimore, Maryland; b Wendy Klag Center for Autism and Developmental Disabilities, Bloomberg School of Public Health, Johns Hopkins University, Baltimore, Maryland; c Department of Mental Health, Bloomberg School of Public Health, Johns Hopkins University, Baltimore, Maryland; d Department of Epidemiology, School of Public Health, University of Michigan, Ann Arbor, Michigan; e Division of Research, Kaiser Permanente Northern California, Pleasanton, California; f Department of Health Systems Science, Kaiser Permanente School of Medicine, Pasadena, California; g Department of Public Health Sciences, School of Medicine, University of California at Davis (UC Davis), Davis, California; h UC Davis MIND (Medical Investigations of Neurodevelopmental Disorders) Institute, UC Davis, Sacramento, California; i AJ Drexel Autism Institute, Drexel University, Philadelphia, Pennsylvania; j College of Health and Human Development, Pennsylvania State University, State College, Pennsylvania; k Rollins School of Public Health, Emory University, Atlanta, Georgia

**Keywords:** Air pollution, Epigenetic aging, DNA methylation, Placenta

## Abstract

**Background:**

Prenatal air pollutants have been associated with adverse birth outcomes, and DNA methylation (DNAm) changes in placenta may contribute to these associations. DNAm-based epigenetic gestational age (GA) estimators are emerging biomarkers for aging/biological age that can reflect early-life exposures and predict long-term health outcomes. We leveraged 103 mother-offspring pairs from the Early Autism Risk Longitudinal Investigation cohort to assess associations between prenatal air pollution and placental epigenetic GA at birth.

**Methods:**

Prenatal air pollution concentrations (NO_2_, O_3_, PM_2.5_, and PM_10_) were estimated from weekly data from monitoring stations near maternal residence and calculated for preconception and pregnancy periods. DNAm from fetal-side placenta samples was measured on Illumina HumanMethylation450 BeadChip. Epigenetic GA was computed using Lee’s robust placenta clock algorithm. GA acceleration/deceleration was the residual of predicted epigenetic GA on chronologic GA, adjusted (intrinsic) or unadjusted (extrinsic) for cell type proportions. We used linear regressions to examine associations between average air pollution levels in each period and GA acceleration/deceleration, and weekly distributed lag models to examine critical exposure windows.

**Results:**

Higher pregnancy average O_3_ and PM_10_ exposures were associated with decelerated intrinsic (β = −0.65 and −0.79) and extrinsic GA (β = −0.69 and −0.74) at birth (per 10-unit increment). Trimester-specific analyses revealed higher O_3_ and PM_10_ exposures in trimesters 2 to 3 associated with decelerated GA at birth. Weekly distributed lag models suggested pregnancy weeks 21 to 31 and 21 to 29 were critical windows of O_3_ and PM_10_ exposures, respectively.

**Conclusions:**

Prenatal air pollution exposures, especially during mid- to late-pregnancy, were associated with lower biological maturity at birth.

**What this study adds**

Despite well-established associations between prenatal ambient air pollution exposures and adverse birth outcomes, the biological mechanisms underlying these associations are not well understood. We assessed the associations of preconception and pregnancy ambient air pollution exposures with placental epigenetic gestational age at birth. We found associations between O_3_ and PM_10_ exposures during pregnancy and decelerated epigenetic gestational age at birth, and identified mid- to late-pregnancy as critical exposure windows for such associations. These findings contribute to the growing evidence of prenatal ambient air pollution-induced fetal programming alterations and reinforce the importance of air pollution exposure prevention during pregnancy.

## Introduction

Prenatal ambient air pollutants are well-established risk factors for adverse outcomes at birth, including low birth weight, preterm birth, and high infant mortality.^1–4^ As postulated by the Developmental Origins of Health and Disease hypothesis, maternal responses to early-life exposures during the sensitive in utero period have long-term effects influencing offspring’s susceptibility to disease into later adulthood.^5–7^ Consistently, various studies demonstrated prolonged adverse health impacts of prenatal exposure to air pollutants into childhood and adolescence.^8–11^ However, biological pathways associated with prenatal air pollutant exposure effects on offspring are not well understood.

The placenta is a highly specialized organ formed during pregnancy that acts as the interface between mother and embryo/fetus, and plays crucial roles in nutrient transfer and in regulating embryonic/fetal growth and development.^12^ Evolving biological and epidemiological studies have identified air pollution-associated placental alterations.^13–15^ Air pollution particles can pass placenta and translocate directly toward fetus,^16,17^ which induces inflammation,^18,19^ oxidative/nitrosative stress,^13,14^ and epigenetic alterations.^15,20,21^ DNA methylation (DNAm), the most extensively studied epigenetic modification, is critical to fetal programming.^22^ DNAm signatures in placenta have higher variability compared with other tissues,^23,24^ and are sensitive to environmental conditions during pregnancy.^24^ Recent epigenome-wide association studies associated prenatal air pollution with global, regional, and locus-specific placental DNAm changes.^20,25,26^ These findings demonstrated the potential of DNAm signatures as promising biomarkers for early-life environmental exposures.^27^

Epigenetic age, a DNAm-based age estimator, has emerged as one of the most efficient biomarkers for biological/developmental age.^28–30^ Epigenetic age estimators are constructed from CpG sites with age-related methylation changes, predict chronologic age with high accuracy in diverse populations and across tissues.^28,31^ Epigenetic age acceleration/deceleration, the discordance between predicted epigenetic and chronologic age, captures disproportionate biologic aging at the individual level and has been associated with age-related diseases/conditions in elderly adults.^32–35^ In newborns, epigenetic gestational age estimators were developed to predict gestational age from DNAm data in cord blood or placenta tissues collected at birth. Gestational age acceleration/deceleration (GAA/GAD), the difference between predicted epigenetic and chronologic gestational age, is thought to reflect neonatal physiological development and epigenetic programming potentially influenced by early-life environmental exposures.^36–39^ Recent studies associated early-life exposures, such as psychosocial conditions,^40,41^ pregnancy risk factors,^42^ and pregnancy complications,^43^ with GAD, which has been implicated with developmental delays in childhood.^40^

Despite links between air pollution and gestational age as well as between gestational age and epigenetics, very few studies have examined the relationship of prenatal air pollution with GAA/GAD, and those that did were examined in cord blood tissues^44–46^ and limited in types of air pollutant assessed.^26^ In this study, we examined associations of prenatal ambient air pollutant (nitrogen dioxide [NO_2_], ozone [O_3_], and particulate matter less than 2.5 and 10 microns in diameter [PM_2.5_, PM_10_]) exposures during preconception and pregnancy periods with placental GAA/GAD at birth and further evaluated critical exposure windows for such associations.

## Methods

### Study design

The Early Autism Risk Longitudinal Investigation (EARLI; 2009–2012) is a prospective cohort study of autism using a high familial likelihood design.^47^ The study enrolled mothers with a biological child previously diagnosed with autism who were no more than 28 weeks in a subsequent pregnancy or were trying to become pregnant. Maternal participants were eligible if they were 18 years or older, competent to communicate in English, and lived within 2 hours of a study site (Philadelphia, Baltimore, San Francisco Bay Area, Sacramento). Enrolled mothers were closely followed for 2–4 study visits during pregnancy, at birth, and after birth. The EARLI study was approved by Human Subjects Institutional Review Boards from each study site. All maternal participants provided written consent.

### Air pollution measurements

Ambient air pollution assignments were based on prospectively recorded maternal residential address 3 months before conception and throughout pregnancy. Standardization and geo-coding were performed using the Tele Atlas US_Geo_2 database and software (Tele Atlas, Inc., Boston, CA, www.geocoded.com). Air quality of NO_2_, O_3_, PM_2.5_, and PM_10_ was derived from the United States Environmental Protection Agency’s Air Quality System data (www.epa.gov/aqs). Weekly air quality data were spatially interpolated using inverse distance-squared weighting of data from up to four closest monitoring stations located within 50 km of participant’s residence; if one or more stations were located within 5 km of a residence, then only data from those stations were used for interpolation. We calculated weekly preconception and pregnancy exposures based on estimates of gestational age and dates of reported residence. We assigned exposure periods based on the gestational age of newborns at birth: preconception period (3 months before pregnancy), and pregnancy period (conception to birth), including the first trimester (days 1–90 of pregnancy), second trimester (days 91–180 of pregnancy), and third trimester (day 181 of pregnancy to birth). For each participant, we calculated the average air pollutant exposure level in each exposure period as the arithmetic mean of weekly exposures, if no more than 10% of weekly exposures were missing in the given exposure period.

### Sample collection and processing

Trained EARLI study staff were present at each delivery. At each study site, using standardized protocols, fetal-side umbilical placental biopsy samples were collected using Baby Tischler Punch Biopsy Forceps shortly after delivery. Collected sample punches were stored at ambient temperature in RNAlater vials (Qiagen Inc., Valencia, CA; Cat. No. 76154) and shipped on the same day to the Johns Hopkins Biological Repository for storage at −190°C until further processing. Genomic DNA was extracted using a Qiagen DNA Midi Kit (Qiagen Inc.) and quantified using a NanoDrop spectrophotometer (ThermoFisher Scientific, Waltham, MA). DNAm at 485,512 CpG loci was measured using the Illumina Infinium HumanMethylation450 BeadChip (450k array) (Illumina, San Diego, CA). For each sample, 1 μg of high-quality genomic DNA was bisulfite treated using the Zymo EZ-96 DNA Methylation Kit (Zymo Research, Irvine, CA; Cat. No. D5004), as per manufacturer’s instructions, including specific modifications for Illumina 450k array processing. A total of 134 fetal-side placental samples were processed together with maternal and paternal blood, cord blood, and maternal-side placental samples in two batches on the 450k array, with the best possible balance on exposures. Raw.idat files were returned to investigators for downstream data processing.

### DNAm data processing and quality control

Since placental samples were processed together with blood samples on the 450k array, we processed their DNAm data together.^20,47^ We used *minfi* package^48^ to import signal intensities of 1155 blood and placental samples with 485,512 probes into R programming environment. Probes with detection *P*-values >0.01 in >10% of samples (N = 636) or noted cross-reactive probes (N = 29,154)^49^ were removed. We checked samples with >10% of probes with detection *P*-values >0.01 (N = 0). Samples with low overall intensities (<11 relative fluorescence units, placenta N = 2) and samples with discordant reported and DNAm predicted sex (placenta N = 2) were excluded. For samples from twin pairs, one of the twins was randomly selected (placenta N = 2 were excluded). In each tissue type, we performed principal component (PC) analysis to assess potential batch effects by inspecting pairwise scatterplots of DNAm beta value PCs and conducting analysis of variance tests with measured technical covariates. We visually inspected DNAm beta value densities by technical covariates.

We used normal-exponential out-of-band method for background subtraction and dye normalization.^50^ After limiting to fetal-side placenta samples and excluding sample with missing sex (N = 1), batch effects of sample plates and array positions were adjusted by empirical Bayes method^51^ with sex as a covariate, implemented in *Combat* function from *sva* package.^52^ DNAm beta values were then normalized using Horvath’s modified beta mixture quantile dilation method, which rescales the distribution of each array to that of a “gold-standard” array, created by forming the mean DNAm value in the largest single training dataset of Horvath’s study.^28^ After normalization, samples with the first 2 DNAm value PCs deviating from corresponding sex subgroups or with outlier DNAm value densities (N = 7) were excluded.

There were 120 fetal-side placental samples with 455,722 probes that passed DNAm quality control (Figure S1; https://links.lww.com/EE/A342). Cell type (syncytiotrophoblast, trophoblasts, Hofbauer, endothelial, stromal, and nucleated red blood cells) proportions were estimated using a reference-based deconvolution method with a reference panel of 19 third trimester placental samples^53^ using constrained projection approach,^54^ implemented in *planet* package.^55^

### Epigenetic gestational age estimation

Epigenetic gestational age was estimated using Lee’s robust placenta clock model, which was unaffected by common pregnancy complications or preterm births.^38,56^ The model was trained by regressing chronologic gestational age on DNAm levels of CpG sites measured on Illumina 450k array using an elastic net regression.^38,56^ The predicted epigenetic gestational age is a weighted average of DNAm levels at 558 CpG sites selected from the model. No required CpG sites were missing in this study.

We considered two measures of GAA/GAD. Intrinsic GAA/GAD was calculated as the residual from a linear regression of epigenetic gestational age on chronologic gestational age, adjusting for estimated cell type proportions. Intrinsic GAA/GAD measures the difference between predicted epigenetic and chronologic gestational age on cellular level, unconfounded by placental cell composition differences.^57,58^ On the other hand, extrinsic GAA/GAD was calculated as the residual from a univariate linear regression of epigenetic gestational age on chronologic gestational age. Extrinsic GAA/GAD incorporates cellular-level differences between predicted epigenetic and chronologic gestational age, and environmental- or age-related changes in placental cell compositions.^57,58^ For both measures, a positive value indicates GAA or faster biological aging relative to chronologic gestational age, whereas a negative value indicates GAD or slower biological aging relative to chronologic gestational age.

### Covariates

We adjusted the following maternal and child characteristics a priori as possible confounders on associations of interests and possible predictors of placental GAA/GAD at birth (Figure S2; https://links.lww.com/EE/A342): maternal age at delivery, maternal race (Asian, Black/African American, White, and other race), maternal ethnicity (Hispanic and non-Hispanic), maternal education (high school or some college, bachelor’s degree, and master’s degree or higher), annual household income (<$30,000, $30,000–49,999, $50,000–74,999, $75,000–99,999, and ≥$100,000), maternal prepregnancy body mass index (in kg/m^2^; underweight/normal [<24.9], overweight [25.0–29.9], obesity [≥30.0]), child sex (male and female), birth season (warm: April–September and cold: October–March), and study site. All covariates information was obtained from the maternal self-reported questionnaire.

### Statistical analysis

We included mother-offspring pairs with prenatal average ambient air pollution measurements in at least one exposure period (preconception, pregnancy, and trimesters 1, 2, and 3) and that passed DNAm quality control in the analysis. Descriptive statistics on maternal and child characteristics were computed and reported as follows: continuous variables as mean (standard deviation [SD]) if symmetrically distributed, median (interquartile range) if skewed, and categorical variables as frequency (proportion). We assessed pairwise correlations of average air pollutant concentrations in each exposure period. We calculated correlations for chronologic and epigenetic gestational age.

Association testing was restricted to participants with complete covariate information. For each pollutant and exposure period, we used a multivariable linear regression to examine the association of average pollutant level with GAA/GAD at birth, adjusting for covariates aforementioned (individual analytic models, Supplementary Methods; https://links.lww.com/EE/A342). For each pollutant, to account for correlations between air pollutant levels across exposure periods, we extended linear regression models by mutually adjusting for average pollutant levels of the same pollutant across exposure periods, adjusting for covariates aforementioned (mutually adjusted analytic models, Supplementary Methods; https://links.lww.com/EE/A342). We performed sensitivity analyses to test the robustness of results (Supplementary Methods; https://links.lww.com/EE/A342).

To identify critical windows of exposures, we used weekly polynomial distributed lag models (DLMs) to examine associations of weekly air pollutants with GAA/GAD at birth, which fit weekly pollutant levels from preconception to pregnancy week 35 (weeks 1–48 of entire preconception and pregnancy period) into one model, concurrently accounted for current and past air pollutant levels, adjusting for covariates aforementioned, implemented in *dlnm* package (Supplementary Methods; https://links.lww.com/EE/A342).^59^ To avoid missingness of weekly pollutant levels due to preterm births, pregnancy weeks 36 to 39 were excluded in this analysis. Various degrees of freedom were examined, and the best-fitting model was selected based on Akaike information criterion statistics and visual inspection of trends for estimated weekly associations. In sensitivity analyses, we restricted samples to full-term births (chronologic gestational age ≥37 weeks at birth) to assess whether effects were consistent in this subgroup. We also considered weekly natural cubic spline DLMs with various degrees of freedom.

Statistical significance was considered as a two-sided *P*-value <0.05. All statistical analyses were conducted in R (version 4.2.0).^60^

## Results

### Descriptive statistics

A total of 103 mother-offspring pairs with prenatal ambient air pollution measurements and that passed DNAm quality control were included in this study (Figure S1; https://links.lww.com/EE/A342). The mean (SD) chronologic and estimated epigenetic gestational age at birth was 39.2 (1.4) and 38.9 (1.1) weeks, respectively (Table 1 and Table S1; https://links.lww.com/EE/A342). The study population was multiracial and ethnic: among included mothers, 64% reported White race, 15% reported Asian race, 11% reported Black/African American race, and 10% reported other race; and 18% of included mothers reported Hispanic ethnicity. The majority of included mothers had a bachelor’s degree or above (58%); had a middle/high annual household income ($75,000–199,000: 53%); and had an underweight/normal prepregnancy body mass index (41%). The majority of included children were males (56%) and born in cold seasons (53%). Samples with GAD were more likely to be female sex, from families with low/middle income, and overweight/obese mothers.

**Table 1.** Maternal and child participants and sample characteristics of the study population, stratified by intrinsic gestational age acceleration or deceleration

Characteristic (mean [SD]/frequency [%])	Overall (N = 103)	Intrinsic gestational age^a^	
		Acceleration (N = 47)	Deceleration (N = 56)
Chronologic gestational age (in weeks)	39.2 (1.4)	39.2 (1.1)	39.2 (1.6)
Epigenetic gestational age (in weeks)	38.9 (1.1)	39.5 (0.8)	38.3 (1.0)
Maternal age at delivery (in years)	34.1 (4.3)	34.4 (4.7)	33.9 (4.1)
Maternal race			
Asian	15 (15%)	7 (16%)	8 (15%)
Black/African American	11 (11%)	7 (16%)	4 (7.3%)
White	63 (64%)	26 (59%)	37 (67%)
Other	10 (10%)	4 (9.1%)	6 (11%)
Maternal Hispanic ethnicity	19 (18%)	7 (15%)	12 (21%)
Maternal education			
High school of some college	42 (42%)	20 (43%)	22 (40%)
Bachelor’s degree	27 (27%)	11 (24%)	16 (29%)
Master’s degree or higher	32 (32%)	15 (33%)	17 (31%)
Annual household income			
<$30,000	11 (11%)	5 (11%)	6 (12%)
$30,000–49,999	18 (19%)	6 (13%)	12 (23%)
$50,000–74,999	17 (18%)	7 (16%)	10 (19%)
$75,000–99,999	19 (20%)	8 (18%)	11 (21%)
≥$100,000	32 (33%)	19 (42%)	13 (25%)
Maternal prepregnancy body mass index			
Underweight or normal (<24.9 kg/m^2^)	40 (41%)	20 (44%)	20 (38%)
Overweight (25–29.9 kg/m^2^)	31 (32%)	13 (29%)	18 (34%)
Obesity (≥30.0 kg/m^2^)	27 (28%)	12 (27%)	15 (28%)
Child female sex	45 (44%)	13 (28%)	32 (57%)
Warm birth season (April–September)	48 (47%)	21 (45%)	27 (48%)
Study site			
Philadelphia	28 (27%)	14 (30%)	14 (25%)
Baltimore	26 (25%)	12 (26%)	14 (25%)
Sacramento	30 (29%)	13 (28%)	17 (30%)
San Francisco Bay Area	19 (18%)	8 (17%)	11 (20%)
Estimated cell type proportions (%)^b^			
Trophoblasts	13.3 (3.9)	12.8 (3.1)	13.7 (4.4)
Stromal cells	11.0 (2.7)	10.8 (2.9)	11.1 (2.5)
Hofbauer cells	2.8 (1.3)	2.8 (1.1)	2.7 (1.4)
Endothelial cells	7.4 (1.9)	7.4 (1.8)	7.5 (2.0)
Nucleated red blood cells	4.3 (1.3)	4.3 (0.8)	4.3 (1.6)
Syncytiotrophoblast	64.5 (6.0)	65.2 (5.8)	63.9 (6.2)
Preconception period average exposure levels			
NO_2_ (ppb)	12.9 (4.0)	13.0 (4.1)	12.9 (3.9)
O_3_ (ppb)	25.3 (7.4)	25.8 (7.3)	24.9 (7.5)
PM_2.5_ (μg/m^3^)	10.0 (2.6)	9.8 (2.7)	10.3 (2.6)
PM_10_ (μg/m^3^)	17.3 (3.9)	17.2 (4.2)	17.4 (3.6)
Pregnancy period average exposure levels			
NO_2_ (ppb)	12.7 (3.4)	12.8 (3.6)	12.6 (3.3)
O_3_ (ppb)	25.8 (3.9)	25.0 (3.6)	26.6 (4.1)
PM_2.5_ (μg/m^3^)	9.7 (1.6)	9.8 (1.6)	9.7 (1.7)
PM_10_ (μg/m^3^)	17.6 (2.9)	16.9 (3.3)	18.2 (2.5)

Missing data: maternal race (N = 4); maternal education (N = 2); annual household income (N = 6); maternal prepregnancy body mass index (N = 5); preconception period average NO_2_, PM_2.5_ level (N = 7); preconception period average O_3_ level (N = 11); preconception average PM_10_ level (N = 8); pregnancy period average NO_2_, PM_2.5_ level (N = 9); pregnancy period average O_3_ level (N = 13); pregnancy period average PM_10_ level (N = 10).

a Intrinsic gestational age acceleration or deceleration was calculated as the residual from a linear regression with epigenetic gestational age as dependent variable and chronologic gestational age as independent variable, adjusting for estimated cell type proportions. Intrinsic gestational age acceleration was defined as a positive residual, and intrinsic gestational age deceleration was defined as a negative or zero residual.

b Cell type proportions were estimated with a reference panel of 19 third trimester human placental samples using constrained projection approach.

Kg/m^2^, kilograms per square meter; NO_2_, nitrogen dioxide; O_3_, ozone; PM_10_, particulate matter less than 10 microns in diameter; PM_2.5_, particulate matter less than 2.5 microns in diameter; ppb, parts per billion; SD, standard deviation; μg/m^3^, microgram per cubic meter of air.

Average (SD) NO_2_, O_3_, PM_2.5_, and PM_10_ levels were 12.9 (4.0) ppb, 25.3 (7.4) ppb, 10.0 (2.6) μg/m^3^, and 17.3 (3.9) μg/m^3^ during preconception, and 12.7 (3.4) ppb, 25.8 (3.9) ppb, 9.7 (1.6) μg/m^3^, and 17.6 (2.9) μg/m^3^ during pregnancy, respectively (Table 1 and Table S1; https://links.lww.com/EE/A342, Figure S3; https://links.lww.com/EE/A342). Average air pollutant levels during pregnancy were higher at East Coast than West Coast sites (Figure S4; https://links.lww.com/EE/A342). Within preconception and pregnancy periods, correlations between average air pollutants were low to moderate (Pearson’s correlation coefficient *r* range: −0.22, 0.63) (Figure S5; https://links.lww.com/EE/A342). Moderate negative correlations between NO_2_ and O_3_ were observed in each trimester during pregnancy (*r* range: −0.57, −0.32). Correlations between average NO_2_, O_3_, PM_2.5_, and PM_10_ across preconception and pregnancy periods were low to moderate (*r* = 0.53, −0.13, −0.02, and 0.25, respectively) (Figure S6; https://links.lww.com/EE/A342). Medium–high correlations were observed between chronologic and epigenetic gestational age (*r* = 0.63) (Figure S7; https://links.lww.com/EE/A342).

### Average air pollution and epigenetic gestational age

For each air pollutant and each exposure period, a total of 83 to 89 participants contributed to the association estimates. The contributing number varied by air pollutant because of slight differences in missingness for average air pollution levels and covariates. For a 10 ppb increase in pregnancy average O_3_ exposure, we observed a 0.65 (95% confidence interval [CI]: 0.11, 1.20) week of intrinsic decelerated gestational aging at birth in an individual analytic model (Table 2). In addition, a 10 μg/m^3^ increase in pregnancy average PM_10_ exposure was associated with a 0.79 (95% CI: 0.17, 1.40) week deceleration of intrinsic gestational aging at birth. Consistently, pregnancy average O_3_ and PM_10_ exposures were associated with decelerated extrinsic gestational aging at birth (β = −0.69, 95% CI: −1.26, −0.11; β = −0.74, 95% CI: −1.42, −0.06; per 10-unit increment, respectively) in individual analytical models. Beta estimates were similar with larger variance in models mutually adjusting for average air pollutant levels during preconception and pregnancy periods (Table S2; https://links.lww.com/EE/A342). We additionally observed a marginal association of preconception average O_3_ exposure with extrinsic accelerated gestational aging at birth (β = 0.26, 95% CI: −0.03, 0.55), which was largely attenuated after mutually adjusting for pregnancy average O_3_ exposure (β = 0.11, 95% CI: −0.22, 0.43), indicating that the association was likely driven by the negative correlation between preconception and pregnancy average O_3_ levels (*r* = −0.13). We did not observe any significant association of preconception and pregnancy average NO_2_ and PM_2.5_ with placental epigenetic gestational age at birth. Associations of pregnancy average O_3_ and PM_10_ exposures with GAD at birth were consistent in sensitivity analyses (Table S4; https://links.lww.com/EE/A342).

**Table 2.** Associations of preconception and pregnancy average ambient air pollution and placental epigenetic gestational age at birth in weeks, results of individual analytic models

Pollutant	N	Intrinsic gestational age acceleration/deceleration^a^		Extrinsic gestational age acceleration/deceleration^a^	
		*β* (95% CI)	*P*-value	*β* (95% CI)	*P*-value
Preconception					
NO_2_ (ppb)	89	−0.24 (−0.78, 0.31)	0.386	−0.29 (−0.87, 0.29)	0.324
O_3_ (ppb)	85	0.17 (−0.11, 0.45)	0.242	0.26 (−0.03, 0.55)	0.083
PM_2.5_ (μg/m^3^)	89	−0.36 (−1.12, 0.40)	0.347	−0.28 (−1.10, 0.54)	0.498
PM_10_ (μg/m^3^)	88	−0.10 (−0.58, 0.38)	0.682	−0.06 (−0.59, 0.46)	0.815
Pregnancy					
NO_2_ (ppb)	87	0.02 (−0.59, 0.63)	0.946	0.14 (−0.52, 0.79)	0.675
O_3_ (ppb)	83	−0.65 (−1.20, −0.11)	0.019	−0.69 (−1.26, −0.11)	0.020
PM_2.5_ (μg/m^3^)	87	−0.36 (−1.95, 1.23)	0.650	0.34 (−1.37, 2.05)	0.694
PM_10_ (μg/m^3^)	86	−0.79 (−1.40, −0.17)	0.013	−0.74 (−1.42, −0.06)	0.035

a Adjusted for maternal age at delivery, maternal race, maternal ethnicity, maternal education, annual household income, maternal prepregnancy body mass index, child sex, birth season, and study site.

CI indicates confidence interval; N, sample size; NO_2_, nitrogen dioxide; O_3_, ozone; PM_10_, particulate matter less than 10 microns in diameter; PM_2.5_, particulate matter less than 2.5 microns in diameter; ppb, parts per billion; μg/m^3^, micrograms per cubic meter of air.

We further partitioned pregnancy periods into trimesters and tested associations of trimester-specific average air pollutant exposures with GAA/GAD at birth. A 10 ppb increase in average O_3_ exposure during the second and third trimesters was associated with a 0.26 (95% CI: 0.01, 0.52) and 0.32 (95% CI: 0.0004, 0.63) week deceleration of intrinsic gestational aging at birth, in individual analytic models, respectively (Table 3). Average PM_10_ exposure during the second and third trimesters was associated with decelerated intrinsic gestational aging at birth (β = −0.50, 95% CI: −0.96, −0.05; β = −0.44, 95% CI: −0.87, −0.01; per 10 μg/m^3^ increment, respectively). Consistently, average O_3_ exposure during the second and third trimesters was associated with extrinsic decelerated gestational aging at birth (β = −0.29, 95% CI: −0.56, −0.02; β = −0.49, 95% CI: −0.83, −0.16; per 10 ppb increment, respectively). Marginal and significant associations with decelerated extrinsic gestational aging at birth were observed for average PM_10_ exposure during the second and third trimesters (β = −0.43, 95% CI: −0.94, 0.08; β = −0.59, 95% CI: −1.06, −0.13; per 10 μg/m^3^ increment, respectively). Beta estimates were attenuated with larger variance after mutually adjusting for average pollutant levels across all three trimesters (Table S3; https://links.lww.com/EE/A342). No significant association was observed for any trimester-specific average NO_2_ or PM_2.5_ levels with placental epigenetic gestational age at birth.

**Table 3.** Associations of trimester-specific average ambient air pollution and placental epigenetic gestational age at birth in weeks, results of individual analytic models

Pollutant	N	Intrinsic gestational age acceleration/deceleration^a^		Extrinsic gestational age acceleration/deceleration^a^	
		*β* (95% CI)	*P*-value	*β* (95% CI)	*P*-value
Trimester 1					
NO_2_ (ppb)	90	−0.36 (−0.83, 0.12)	0.143	−0.30 (−0.82, 0.22)	0.251
O_3_ (ppb)	88	0.09 (−0.34, 0.51)	0.689	0.21 (−0.23, 0.65)	0.346
PM_2.5_ (μg/m^3^)	90	−0.13 (−1.06, 0.80)	0.786	0.35 (−0.64, 1.35)	0.480
PM_10_ (μg/m^3^)	89	−0.17 (−0.59, 0.24)	0.405	−0.04 (−0.49, 0.41)	0.854
Trimester 2					
NO_2_ (ppb)	89	0.12 (−0.31, 0.55)	0.576	0.29 (−0.17, 0.75)	0.220
O_3_ (ppb)	87	−0.26 (−0.52, −0.01)	0.044	−0.29 (−0.56, −0.02)	0.039
PM_2.5_ (μg/m^3^)	90	−0.49 (−1.24, 0.26)	0.197	−0.12 (−0.94, 0.70)	0.774
PM_10_ (μg/m^3^)	89	−0.50 (−0.96, −0.05)	0.031	−0.43 (−0.94, 0.08)	0.094
Trimester 3					
NO_2_ (ppb)	92	0.22 (−0.22, 0.66)	0.315	0.34 (−0.14, 0.82)	0.158
O_3_ (ppb)	90	−0.32 (−0.63, −0.0004)	0.0497	−0.49 (−0.83, −0.16)	0.004
PM_2.5_ (μg/m^3^)	92	0.01 (−0.63, 0.66)	0.965	−0.06 (−0.76, 0.65)	0.868
PM_10_ (μg/m^3^)	92	−0.44 (−0.87, −0.01)	0.046	−0.59 (−1.06, −0.13)	0.013

a Adjusted for maternal age at delivery, maternal race, maternal ethnicity, maternal education, annual household income, maternal prepregnancy body mass index, child sex, birth season, and study site.

CI indicates confidence interval; N, sample size; NO_2_, nitrogen dioxide; O_3_, ozone; PM_10_, particulate matter less than 10 microns in diameter; PM_2.5_, particulate matter less than 2.5 microns in diameter; ppb, parts per billion; μg/m^3^, micrograms per cubic meter of air.

### Critical windows of exposure

**Figure 1.**
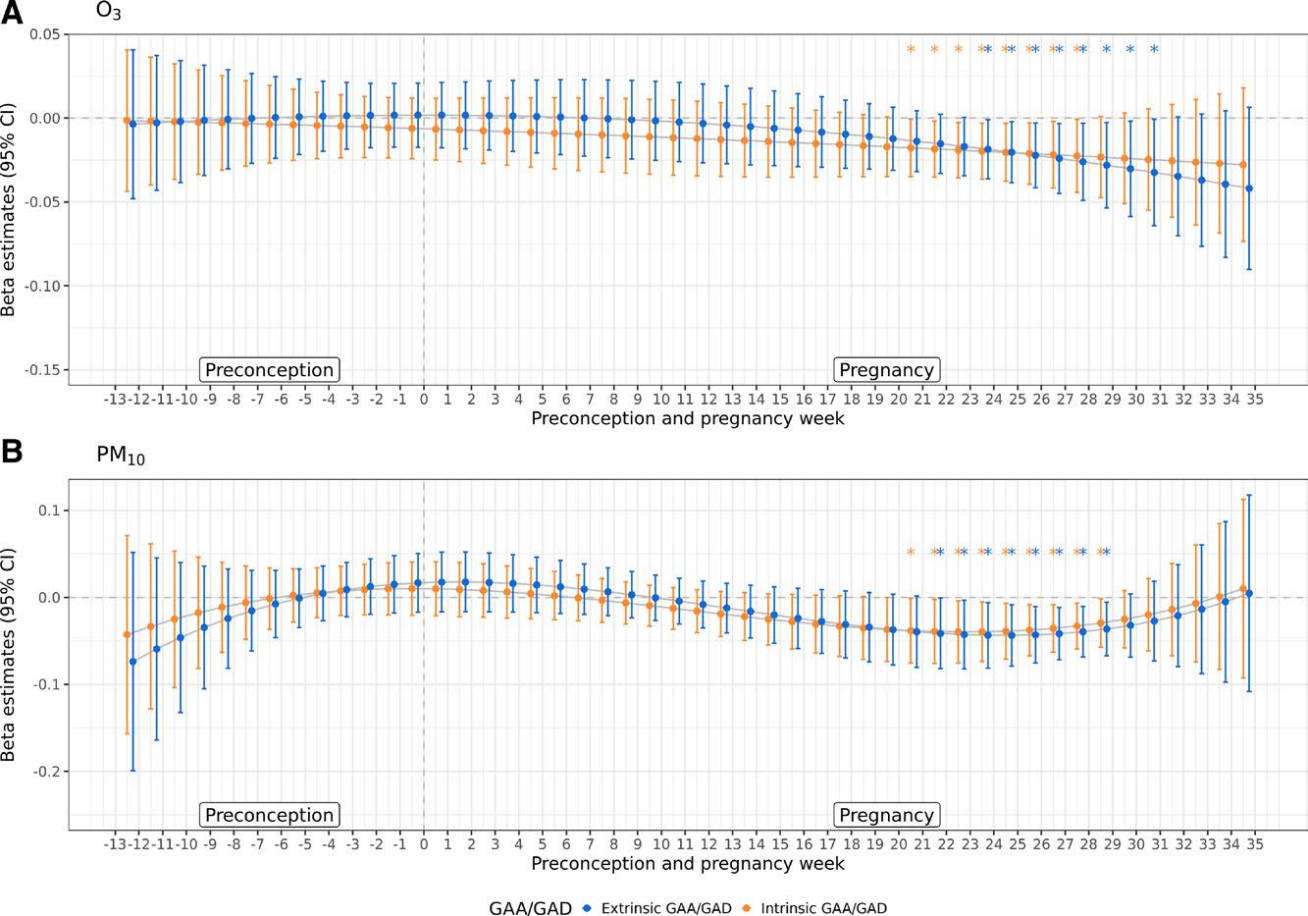
Adjusted associations for prenatal weekly ambient air pollution and placental epigenetic gestational age at birth, with polynomial distributed lag models for (A) O_3_ (n samples = 82) and (B) PM_10_ (n samples = 85). CI indicates confidence interval; O_3_, ozone; PM_10_, particulate matter less than 10 microns in diameter.

In polynomial weekly DLMs, associations with intrinsic and extrinsic decelerated gestational aging at birth were observed for O_3_ exposures during pregnancy weeks 21 to 28 and 24 to 31, and for PM_10_ exposures during pregnancy weeks 21 to 29 and 22 to 29, respectively (Figure 1). Results were consistent with trimester-specific analyses suggesting associations with decelerated gestational aging at birth for average O_3_ and PM_10_ exposures during trimesters 2 and 3. The strongest associations with intrinsic and extrinsic decelerated gestational aging at birth were observed for O_3_ exposure during pregnancy week 28 and 31 (β = −0.02, 95% CI: −0.04, −0.0008; β = −0.03, 95% CI: −0.06, −0.0006; per 10 ppb increment, respectively), and for PM_10_ exposure during pregnancy week 23 and 25 (β = −0.04, 95% CI: −0.08, −0.004; β = −0.04, 95% CI: −0.08, −0.008; per 10 μg/m^3^ increment, respectively). We additionally found associations of NO_2_ exposures during late pregnancy (weeks 29–32) with accelerated gestational aging at birth (Figure S8; https://links.lww.com/EE/A342). No significant association was observed for PM_2.5_ exposure in either preconception or pregnancy week with placental epigenetic gestational age at birth.

Associations were consistent in sensitivity analysis using natural cubic spline weekly DLMs, which suggested associations with decelerated gestational aging at birth for O_3_ and PM_10_ exposures during pregnancy weeks 21 to 32 and 23 to 28, respectively (Figure S9; https://links.lww.com/EE/A342). In addition, we observed associations between NO_2_ exposures during pregnancy weeks 26 to 30 and accelerated gestational aging at birth (Figure S10; https://links.lww.com/EE/A342). Associations with decelerated gestational aging at birth were observed for O_3_ and PM_10_ exposures during pregnancy weeks 25 to 29 and 27 to 31, respectively, in sensitivity analyses of polynomial or natural cubic spline weekly DLMs limited to 95 full-term birth samples (Figures S11–S14; https://links.lww.com/EE/A342).

## Discussion

In this cohort study of 103 mother-offspring pairs, we found associations of pregnancy O_3_ and PM_10_ exposures with decelerated gestational aging at birth in placenta, suggesting lower biological maturity. Trimester-specific analyses revealed associations between O_3_ and PM_10_ exposures during the second and third trimesters with decelerated gestational aging at birth. Weekly DLMs suggested associations with decelerated gestational aging at birth for O_3_ and PM_10_ exposures during mid- to late-pregnancy.

We found associations of ambient air average O_3_ and PM_10_ exposures during pregnancy with decelerated gestational aging at birth, captured by DNAm predicted biological aging in placenta. This is consistent with recent studies showing prenatal air pollution-associated changes in biological aging captured by alternative aging-related biomarkers in cord blood and placenta, including telomere length^60–64^ and mitochondrial DNA content.^65–67^ The associations were also consistent with studies assessing prenatal air pollution and DNAm signatures in cord blood and placenta, which suggested prenatal air pollution associated DNAm alterations of genes involved in growth and embryonal development, as well as biological aging pathways.^21,25,68–71^ Our findings contribute to the growing evidence of air pollution-induced fetal programming alterations at cellular levels, as captured by epigenetic aging in placenta.

We identified mid- to late-pregnancy as critical windows for O_3_ and PM_10_ exposures, which aligns with changes in maternal-embryonic/fetal interface throughout pregnancy: during the first month, the blood-placental barrier is thick and lacks maternal blood perfusion, and it thins as pregnancy progresses to allow maternal blood supply, fetal capillary development, blood perfusion, and nutrients exchange.^3^ Air pollutants translocated through the maternal-fetal interface may induce inflammation and oxidative stress, impairing placenta barrier efficacy.^3,15,72^ Mid- to late-pregnancy is characterized by fast somatic growth and neurodevelopment. Air pollution exposures during this vulnerable period may critically impact the biological maturity of offspring. The critical exposure window we found is consistent with a study on placental telomere length,^60^ but contrasts with our previous findings in cord blood, which suggests early and mid-pregnancy as critical exposure periods for GAD.^46^ Further investigation is warranted to elucidate the critical time window and tissue-specific biological mechanisms of prenatal air pollution exposures with biological aging of offspring. We additionally observed associations between NO_2_ exposures during pregnancy weeks 29 to 32 and GAA at birth, overlapping with the critical window for O_3_ exposure; however, the directions of associations were opposite. This aligns with inverse correlations of pregnancy NO_2_ and O_3_ exposures in this study and commonly observed in previous studies.^73–75^

We used both intrinsic and extrinsic GAA/GAD to measure epigenetic aging. Intrinsic epigenetic age acceleration/deceleration is conceived to reflect “pure” epigenetic aging at a cellular level, unconfounded by cell type composition differences. It is often more correlated with chronologic age, relatively stable, and less susceptible to external environment changes.^76^ In contrast, extrinsic epigenetic age acceleration/deceleration is influenced by external environment and, thus, may be a more sensible biomarker for environmental exposures.^77^ Interestingly, despite a large portion of overlap for critical windows for intrinsic and extrinsic GAD, late-pregnancy weeks may be a more critical period for extrinsic compared to intrinsic GAD. This may reflect accumulated environmentally induced cellular-level DNAm changes^78^ and dynamic changes in cell counts in placenta throughout pregnancy.^53,79,80^ Residual confounding from imprecise cell type deconvolution may also contribute to findings, as the current reference panel is relatively small and has limited available cell types.^53^ Nevertheless, associations with extrinsic GAD for air pollution near delivery were likely driven by preterm births, as no associations were observed for air pollution after pregnancy week 34 in sensitivity analysis of uncomplicated term pregnancy samples.

The placenta is easily assessable at delivery, and sample collection is noninvasive. Moreover, compared to messenger RNA and protein, DNAm in placenta is more stable and less susceptible to technological variations.^81^ These features enable placental epigenetic gestational age as a promising biomarker of fetal biological maturity, whose acceleration/deceleration has the potential to quantify early-life exposures and predict adverse outcomes. GAD is linked to early childhood psychiatric problems,^40,42^ while GAA is associated with increased birth weight,^82,83^ birth length,^82,83^ and fetal head circumference.^83,84^ However, evidence is limited regarding the influence of GAA/GAD at birth on disease onset and progression later in life. In addition, most studies measured epigenetic gestational age in cord blood, while research on placental epigenetic gestational age and health outcomes is limited. Cross-tissue analysis suggested that factors influencing GAA/GAD are tissue-specific.^85^ Future studies to elucidate health outcomes of placental GAA/GAD into childhood and adolescence are warranted.

To our knowledge, this is the first study to assess associations of multiple prenatal air pollutants with placental epigenetic gestational age at birth. We used a well-characterized prospective cohort with data on weekly air pollution throughout preconception and pregnancy, and placental DNAm measurements. Findings were consistent across multiple analytic models and sensitivity analyses, suggesting robustness of evidence.

We acknowledge the following limitations: First, we did not account for indoor/traffic-related air pollutants during preconception/pregnancy, which may bias association estimates.^45^ Second, we did not account for correlations of different air pollutants within the same exposure period. However, associations of O_3_ and PM_10_ exposures with GAD at birth are unlikely to be driven by correlations between pollutants, given their mild correlations during pregnancy. Third, since EARLI is an autism cohort using a high familial likelihood design, newborns in this study are at higher risks for autism or developmental delays,^86^ which may influence the generalizability of findings. Fourth, sex differences of GAA/GAD related to pregnancy complications or fetal growth were previously reported.^43,84^ However, given the limited sample size of sex subgroups, we decided a priori not to examine sex-specific associations. Given the differences in fetal development patterns by sex, future exploration of sex-specific associations between prenatal air pollution and epigenetic gestational age is warranted. Fifth, spatial resolutions of air pollution estimates, as we used here based on residential address, may not be as finely resolved as estimates provided by more recently developed spatial-temporal prediction models, which incorporates information from multiple sources and at greater resolution.^87^ Sixth, we are subject to biases common in observational studies, including unmeasured confounders, information bias of self-reported covariates, and missing not at random. Finally, association estimates may lack precision due to modest sample size. Replication in future studies with larger sample sizes or integrating data from multiple studies through cross-cohort mixture analysis is needed to assess the robustness of the associations and precisely estimate the effect sizes.^88^

## Conclusions

Prenatal exposures to O_3_ and PM_10_, especially from mid- to late-pregnancy, were associated with placental decelerated gestational aging at birth. Findings contribute to the growing evidence of epigenetic aging responsive to pregnancy environmental exposures, which has the potential for long-term health and developmental impacts, and reinforce the importance of air pollution exposure mitigation during pregnancy. Future studies are needed to further understand epigenetic mechanisms involved in fetal programming responsive to environmental exposures and downstream health outcomes in offspring.

## Conflicts of interest statement

The authors declare that they have no conflicts of interest with regard to the content of this report.

## Acknowledgments

The author would like to thank the study participants and their parents in the EARLI study, the laboratory staff at the Johns Hopkins Biological Repository, as well as the field team for their contributions to the EARLI study.

## Supplementary Material
