# Supplementary material for: The association of prenatal ambient air pollution with placental epigenetic gestational age at birth
Source: Environ Epidemiol. 2025 May 5;9(3):e384. doi: 10.1097/EE9.0000000000000384 (PMC12055125; doi:10.1097/EE9.0000000000000384)
Supplement: Supplementary file 1 [file ee9-9-e384-s001.pdf]

The association of prenatal ambient air pollution with placental epigenetic gestational age at birth

Supplemental Digital Content

Supplementary Methods

Equations for statistical analysis

1. Individual analytic models: for each air pollutant (AP) and each exposure period,

$$\text{E(GAA/GAD)} = \alpha + \beta \cdot \text{average AP}_{\text{exposure period}} + \boldsymbol{\gamma} \cdot \mathbf{Z}$$

where β coefficients are estimated differences in intrinsic or extrinsic gestational age acceleration/deceleration (GAA/GAD) for a 10-unit difference of the average pollutant level, and $\mathbf{Z}$ is the covariates matrix.

2. Mutually adjusted analytic models: for each AP,

$$\text{E(GAA/GAD)} = \alpha + \sum_{\text{exposure period i}} \beta_i \cdot \text{average AP}_{\text{exposure period i}} + \boldsymbol{\gamma} \cdot \mathbf{Z}$$

where β coefficients are estimated differences in intrinsic or extrinsic GAA/GAD for a 10-unit difference of the average pollutant level, and $\mathbf{Z}$ is the covariates matrix. We performed the analysis mutually adjusted for average AP in preconception and pregnancy exposure periods, as well as mutually adjusted for average AP in trimester 1, trimester 2, and trimester 3 exposure periods.

3. Weekly distributed lag models (DLM): for each AP,

$$\text{E(GAA/GAD)} = \alpha + \sum_{t=0}^{47} s_t(\beta_t, \text{weekly AP}_t) + \boldsymbol{\gamma} \cdot \mathbf{Z}$$

where weekly AP_t is a series of weekly AP measured at 0, 1, 2, …, 47 week(s) prior to pregnancy week 35, corresponding to pregnancy week 35, 34, 33, …, preconception week 1; the functions s_t specify the

relationships between AP measures and the predictor (vectors β_t); [1] and $\mathbf{Z}$ is the covariates matrix. In primary analyses, we specified the effect of AP as linear, and the lagged effect of AP as polynomial (polynomial DLM): $s_t(\beta_t, \text{weekly AP}_t) = \sum_{k=0}^{K} \beta_k \text{AP}_t^k$. In sensitivity analyses, we specified the effect of AP as linear, and the lagged effect of AP as natural cubic spline (natural cubic spline DLM): $s_t(\beta_t, \text{weekly AP}_t) = \sum_{k=0}^{K} \beta_k B_k(\text{AP}_t)$, where B_k are the natural cubic spline basis functions.

Genotyping and genetics data processing

Genetic data was measured using the Illumina Infinium Omni5Exome-4 Kit at the Johns Hopkins University Center of Inherited Disease Research (CIDR). Samples of 841 EARLI families (including maternal, paternal, proband, and infant samples) from 254 families and 18 HapMap control samples were processed together, with 4.6 million single nucleotide polymorphisms (SNPs) measured. Probes with technical problems flagged by CIDR or missing genomic location information were excluded. SNPs with minor allele frequencies > 5% were excluded if they had a missingness rate > 5%. SNPs with minor allele frequencies < 5% were excluded if they had a missingness rate > 1%. After quality control, there were 2.5 million SNPs for 827 samples, which were further merged with 1000 genomes project (1000GP, version 5) data [2]. Using the *SmartPCA* program from the *EIGENSOFT* package (version 6.1.4), maternal genetic principal components were generated to model ancestry variation [3,4].

Sensitivity analysis

We performed the following sensitivity analyses to test robustness of our results: (1) We adjusted for East or West coast region instead of study sites to assess influence of geographical regions on the association of interests; (2) Given that self-reported race and ethnicity are influenced by a combination of genetic ancestry and social recognitions,[5] we adjusted for first 5 maternal genetic PCs instead of self-reported

races and ethnicities to assess its influence on the association of interests. (3) To assess influence of missing data, assuming missing at random, we used multiple imputation with chained equations (R package *mice*) to impute missing exposures and covariates information (average pollutant levels in preconception and pregnancy periods, maternal race, maternal education, annual household income, maternal pre-pregnancy body mass index). We generated 20 complete datasets and combined results according to Rubin's rules.[6,7]

References:

1. Patterson N, Price AL, Reich D. Population structure and eigenanalysis. 2006;2(12):e190.

2. Gasparrini A. Distributed lag linear and non-linear models in R: the package dlnm. 2011;43(8):1.

3. Mersha TB, Abebe T. Self-reported race/ethnicity in the age of genomic research: its potential impact on understanding health disparities. 2015;9:1–15.

4. White IR, Royston P, Wood AM. Multiple imputation using chained equations: issues and guidance for practice. 2011;30(4):377–99.

5. Consortium 1000 Genomes Project. An integrated map of genetic variation from 1,092 human genomes. 2012;491(7422):56.

6. Rubin DB. Multiple imputation after 18 years. 1996;91(434):473–89.

7. Price AL, Patterson NJ, Plenge RM, Weinblatt ME, Shadick NA, Reich D. Principal components analysis corrects for stratification in genome-wide association studies. 2006;38(8):904–9.

Table S1. Maternal and child participants and sample characteristics of the study population, stratified by extrinsic gestational age acceleration or deceleration.

Characteristic (mean (SD) / frequency (%))	Overall (N = 103)	Extrinsic gestational age†	
		Acceleration (N = 49)	Deceleration (N = 54)
**Chronologic gestational age (in weeks)**	39.2 (1.4)	39.3 (1.1)	39.1 (1.7)
**Epigenetic gestational age (in weeks)**	38.9 (1.1)	39.5 (0.7)	38.2 (1.0)
**Maternal age at delivery (in years)**	34.1 (4.3)	34.5 (4.6)	33.8 (4.1)
**Maternal race**			
Asian	15 (15%)	6 (13%)	9 (17%)
Black/African American	11 (11%)	6 (13%)	5 (9.4%)
White	63 (64%)	30 (65%)	33 (62%)
Other	10 (10%)	4 (8.7%)	6 (11%)
**Maternal Hispanic ethnicity**	19 (18%)	8 (16%)	11 (20%)
**Maternal education**			
High school of some college	42 (42%)	20 (42%)	22 (42%)
Bachelor's degree	27 (27%)	12 (25%)	15 (28%)
Master's degree or higher	32 (32%)	16 (33%)	16 (30%)
**Annual household income**			
<\$30,000	11 (11%)	4 (8.5%)	7 (14%)
\$30,000–49,999	18 (19%)	6 (13%)	12 (24%)
\$50,000–74,999	17 (18%)	8 (17%)	9 (18%)
\$75,000–99,999	19 (20%)	9 (19%)	10 (20%)
≥\$100,000	32 (33%)	20 (43%)	12 (24%)
**Maternal pre-pregnancy body mass index**			
Underweight or normal (<24.9 kg/m^2)	40 (41%)	21 (46%)	19 (37%)
Overweight (25–29.9 kg/m^2)	31 (32%)	14 (30%)	17 (33%)
Obesity (≥30.0 kg/m^2)	27 (28%)	11 (24%)	16 (31%)
**Child female sex**	45 (44%)	13 (27%)	32 (59%)
**Warm birth season (April to September)**	48 (47%)	21 (43%)	27 (50%)
**Study site**			
Philadelphia	28 (27%)	14 (29%)	14 (26%)
Baltimore	26 (25%)	12 (24%)	14 (26%)
Sacramento	30 (29%)	16 (33%)	14 (26%)
San Francisco Bay Area	19 (18%)	7 (14%)	12 (22%)
**Estimated cell type proportions (%)§**			
Trophoblasts	13.3 (3.9)	12.2 (3.3)	14.4 (4.0)
Stromal cells	11.0 (2.7)	10.5 (2.4)	11.4 (2.8)
Hofbauer cells	2.8 (1.3)	2.7 (1.2)	2.8 (1.4)
Endothelial cells	7.4 (1.9)	7.4 (1.7)	7.4 (2.1)
Nucleated red blood cells	4.3 (1.3)	4.2 (1.0)	4.4 (1.5)
Syncytiotrophoblast	64.5 (6.0)	66.2 (5.9)	63.0 (5.8)
**Preconception period average exposure levels**			
NO_2 (ppb)	12.9 (4.0)	13.1 (4.3)	12.8 (3.8)
O_3 (ppb)	25.3 (7.4)	25.1 (7.0)	25.5 (7.8)
$PM_{2.5}$ (μg/m^3)	10.0 (2.6)	9.8 (2.6)	10.3 (2.7)
PM_{10} (μg/m^3)	17.3 (3.9)	17.0 (4.1)	17.7 (3.7)
**Pregnancy period average exposure levels**			
NO_2 (ppb)	12.7 (3.4)	12.9 (3.6)	12.5 (3.3)
O_3 (ppb)	25.8 (3.9)	25.1 (4.0)	26.5 (3.8)

$PM_{2.5}$ (μg/m^3)	9.7 (1.6)	9.8 (1.5)	9.7 (1.7)
PM_{10} (μg/m^3)	17.6 (2.9)	17.1 (3.2)	18.1 (2.5)

Abbreviations: SD, standard deviation; %, percentage; kg/m^2, kilogram/meter^2; NO_2, nitrogen dioxide; O_3, ozone; $PM_{2.5}$, particulate matter less than 2.5 microns in diameter; PM_{10}, particulate matter less than 10 microns in diameter; ppb, parts per billion; μg/m^3, microgram per cubic meter of air.

Missing data: maternal race (N = 4); maternal education (N = 2); annual household income (N = 6); maternal pre-pregnancy body mass index (N = 5); preconception period average NO_2, $PM_{2.5}$ level (N = 7); preconception period average O_3 level (N = 11); preconception average PM_{10} level (N = 8); pregnancy period average NO_2, $PM_{2.5}$ level (N = 9); pregnancy period average O_3 level (N = 13); pregnancy period average PM_{10} level (N = 10).

[†]Extrinsic gestational age acceleration or deceleration was calculated as the residual from a univariate linear regression with epigenetic gestational age as dependent variable and chronologic gestational age as independent variable. Extrinsic gestational age acceleration was defined as a positive residual, and extrinsic gestational age deceleration was defined as a negative or zero residual.

[§]Cell type proportions were estimated with a reference panel of 19 third trimester human placental samples using constrained projection approach.

Table S2. Association of preconception and pregnancy average ambient air pollution and placental epigenetic gestational age at birth, results of mutually adjusted analytic models.

Pollutant	N	Intrinsic gestational age acceleration/deceleration†		Extrinsic gestational age acceleration/deceleration†	
		β **(95% CI)**	**p-value**	***β*** **(95% CI)**	**p-value**
**Preconception**					
NO_2 (ppb)	86	-0.30 (-0.97, 0.36)	0.368	-0.49 (-1.20, 0.22)	0.173
O_3 (ppb)	81	0.01 (-0.30, 0.32)	0.947	0.11 (-0.22, 0.43)	0.514
$PM_{2.5}$ ($\mu g/m^3$)	86	-0.44 (-1.22, 0.33)	0.260	-0.27 (-1.11, 0.57)	0.517
PM_{10} ($\mu g/m^3$)	85	0.16 (-0.34, 0.65)	0.528	0.14 (-0.40, 0.69)	0.604
**Pregnancy**					
NO_2 (ppb)	86	0.22 (-0.52, 0.96)	0.552	0.45 (-0.34, 1.24)	0.260
O_3 (ppb)	81	-0.70 (-1.33, -0.07)	0.031	-0.63 (-1.30, 0.04)	0.065
$PM_{2.5}$ ($\mu g/m^3$)	86	-0.52 (-2.15, 1.11)	0.523	0.24 (-1.53, 2.01)	0.787
PM_{10} ($\mu g/m^3$)	85	-0.84 (-1.52, -0.16)	0.017	-0.80 (-1.56, -0.05)	0.038

Abbreviations: N, sample size; CI, confidence interval; NO_2, nitrogen dioxide; O_3, ozone; $PM_{2.5}$, particulate matter less than 2.5 microns in diameter; PM_{10}, particulate matter less than 10 microns in diameter; ppb, parts per billion; $\mu g/m^3$, micrograms per cubic meter of air.

†One linear regression model was run for each ambient air pollutant, mutually adjusted for average levels of that pollutant in both preconception and pregnancy periods, and adjusted for covariates including maternal age at delivery, maternal race, maternal ethnicity, maternal education, annual household income, maternal pre-pregnancy body mass index, child sex, birth season, and study site.

Table S3. Association of trimester-specific average ambient air pollution and placental epigenetic gestational age at birth, results of mutually adjusted analytic models.

Pollutant	N	Intrinsic gestational age acceleration/deceleration†		Extrinsic gestational age acceleration/deceleration†	
		β **(95% CI)**	**p-value**	***β*** **(95% CI)**	**p-value**
**Trimester 1**					
NO_2 (ppb)	85	-0.38 (-0.90, 0.15)	0.154	-0.43 (-0.99, 0.14)	0.134
O_3 (ppb)	85	-0.15 (-0.62, 0.32)	0.530	-0.08 (-0.57, 0.41)	0.749
$PM_{2.5}$ ($\mu g/m^3$)	89	0.15 (-0.88, 1.19)	0.771	0.65 (-0.47, 1.77)	0.252
PM_{10} ($\mu g/m^3$)	88	-0.10 (-0.55, 0.35)	0.668	-0.05 (-0.54, 0.44)	0.841
**Trimester 2**					
NO_2 (ppb)	85	0.11 (-0.49, 0.72)	0.709	0.34 (-0.31, 0.99)	0.298
O_3 (ppb)	85	-0.15 (-0.47, 0.16)	0.333	-0.09 (-0.41, 0.24)	0.601
$PM_{2.5}$ ($\mu g/m^3$)	89	-0.49 (-1.27, 0.30)	0.219	-0.08 (-0.93, 0.77)	0.851
PM_{10} ($\mu g/m^3$)	88	-0.29 (-0.87, 0.29)	0.326	-0.13 (-0.77, 0.50)	0.678
**Trimester 3**					
NO_2 (ppb)	85	0.20 (-0.41, 0.81)	0.517	0.11 (-0.55, 0.77)	0.732
O_3 (ppb)	85	-0.29 (-0.75, 0.16)	0.205	-0.48 (-0.95, 0.00)	0.049
$PM_{2.5}$ ($\mu g/m^3$)	89	-0.07 (-0.76, 0.63)	0.850	-0.13 (-0.88, 0.62)	0.726
PM_{10} ($\mu g/m^3$)	88	-0.37 (-0.89, 0.14)	0.153	-0.59 (-1.16, -0.03)	0.040

Abbreviations: N, sample size; CI, confidence interval; NO_2, nitrogen dioxide; O_3, ozone; $PM_{2.5}$, particulate matter less than 2.5 microns in diameter; PM_{10}, particulate matter less than 10 microns in diameter; ppb, parts per billion; $\mu g/m^3$, micrograms per cubic meter of air.

†One linear regression model was run for each ambient air pollutant, mutually adjusting for average levels of that pollutant in trimester 1, trimester 2, and trimester 3 periods, and adjusted for covariates including maternal age at delivery, maternal race, maternal ethnicity, maternal education, annual household income, maternal pre-pregnancy body mass index, child sex, birth season, and study site.

Table S4. Association of preconception and pregnancy average ambient air pollution and placental epigenetic gestational age at birth, results of sensitivity analyses.

Pollutant	N	Intrinsic gestational age acceleration/deceleration†		Extrinsic gestational age acceleration/deceleration†	
		**β (95% CI)**	**p-value**	**β (95% CI)**	**p-value**
**Adjusted for East or West coast region (instead of study sites)**					
**Preconception**					
NO_2 (ppb)	89	-0.27 (-0.81, 0.28)	0.334	-0.32 (-0.91, 0.26)	0.272
O_3 (ppb)	85	0.16 (-0.12, 0.44)	0.256	0.25 (-0.04, 0.54)	0.093
$PM_{2.5}$ ($\mu g/m^3$)	89	-0.45 (-1.19, 0.29)	0.225	-0.41 (-1.21, 0.39)	0.314
PM_{10} ($\mu g/m^3$)	88	-0.09 (-0.56, 0.39)	0.717	-0.05 (-0.58, 0.47)	0.840
**Pregnancy**					
NO_2 (ppb)	87	0.04 (-0.56, 0.63)	0.904	0.19 (-0.45, 0.83)	0.560
O_3 (ppb)	83	-0.66 (-1.19, -0.13)	0.015	-0.73 (-1.29, -0.16)	0.012
$PM_{2.5}$ ($\mu g/m^3$)	87	-0.43 (-2.00, 1.14)	0.587	0.25 (-1.45, 1.95)	0.771
PM_{10} ($\mu g/m^3$)	86	-0.69 (-1.29, -0.09)	0.025	-0.62 (-1.28, 0.05)	0.070
**Adjusted for maternal genetic principal components (instead of self-reported races and ethnicities)**					
**Preconception**					
NO_2 (ppb)	89	-0.20 (-0.74, 0.33)	0.457	-0.38 (-0.97, 0.20)	0.198
O_3 (ppb)	85	0.07 (-0.20, 0.33)	0.616	0.21 (-0.08, 0.50)	0.147
$PM_{2.5}$ ($\mu g/m^3$)	89	-0.09 (-0.84, 0.66)	0.816	-0.02 (-0.84, 0.81)	0.969
PM_{10} ($\mu g/m^3$)	88	-0.11 (-0.55, 0.33)	0.620	-0.09 (-0.59, 0.41)	0.718
**Pregnancy**					
NO_2 (ppb)	87	-0.07 (-0.65, 0.51)	0.812	-0.05 (-0.68, 0.58)	0.872
O_3 (ppb)	83	-0.59 (-1.11, -0.07)	0.027	-0.59 (-1.16, -0.03)	0.040
$PM_{2.5}$ ($\mu g/m^3$)	87	-0.48 (-1.99, 1.03)	0.527	0.17 (-1.49, 1.82)	0.842
PM_{10} ($\mu g/m^3$)	86	-0.68 (-1.29, -0.07)	0.030	-0.69 (-1.37, -0.01)	0.045
**Multiple imputation on missing exposures and covariates information**					
**Preconception**					

NO_2 (ppb)	103	-0.17 (-0.65, 0.30)	0.470	-0.04 (-0.56, 0.49)	0.887
O_3 (ppb)	103	0.11 (-0.11, 0.33)	0.334	-0.50 (-0.93, -0.06)	0.025
$PM_{2.5}$ ($\mu g/m^3$)	103	-0.13 (-0.72, 0.47)	0.670	-0.35 (-1.80, 1.10)	0.633
PM_{10} ($\mu g/m^3$)	103	-0.17 (-0.61, 0.27)	0.452	-0.83 (-1.32, -0.33)	0.001
**Pregnancy**					
NO_2 (ppb)	103	-0.14 (-0.69, 0.40)	0.605	0.13 (-0.46, 0.72)	0.657
O_3 (ppb)	103	0.14 (-0.12, 0.39)	0.281	-0.49 (-0.97, -0.002)	0.049
$PM_{2.5}$ ($\mu g/m^3$)	103	0.12 (-0.58, 0.81)	0.739	0.42 (-1.20, 2.05)	0.607
PM_{10} ($\mu g/m^3$)	103	-0.09 (-0.59, 0.42)	0.737	-0.61 (-1.20, -0.02)	0.041

Abbreviations: N, sample size; CI, confidence interval; NO_2, nitrogen dioxide; O_3, ozone; $PM_{2.5}$, particulate matter less than 2.5 microns in diameter; PM_{10}, particulate matter less than 10 microns in diameter; ppb, parts per billion; $\mu g/m^3$, micrograms per cubic meter of air.

†Separate linear regression models were run for each individual pollutant based on the average ambient air pollutant level for each exposure period. Unless otherwise stated, models were adjusted for maternal age at delivery, maternal race, maternal ethnicity, maternal education, annual household income, maternal pre-pregnancy body mass index, child sex, birth season, and study site.

Figure S1. Flow diagram of sample selection process.

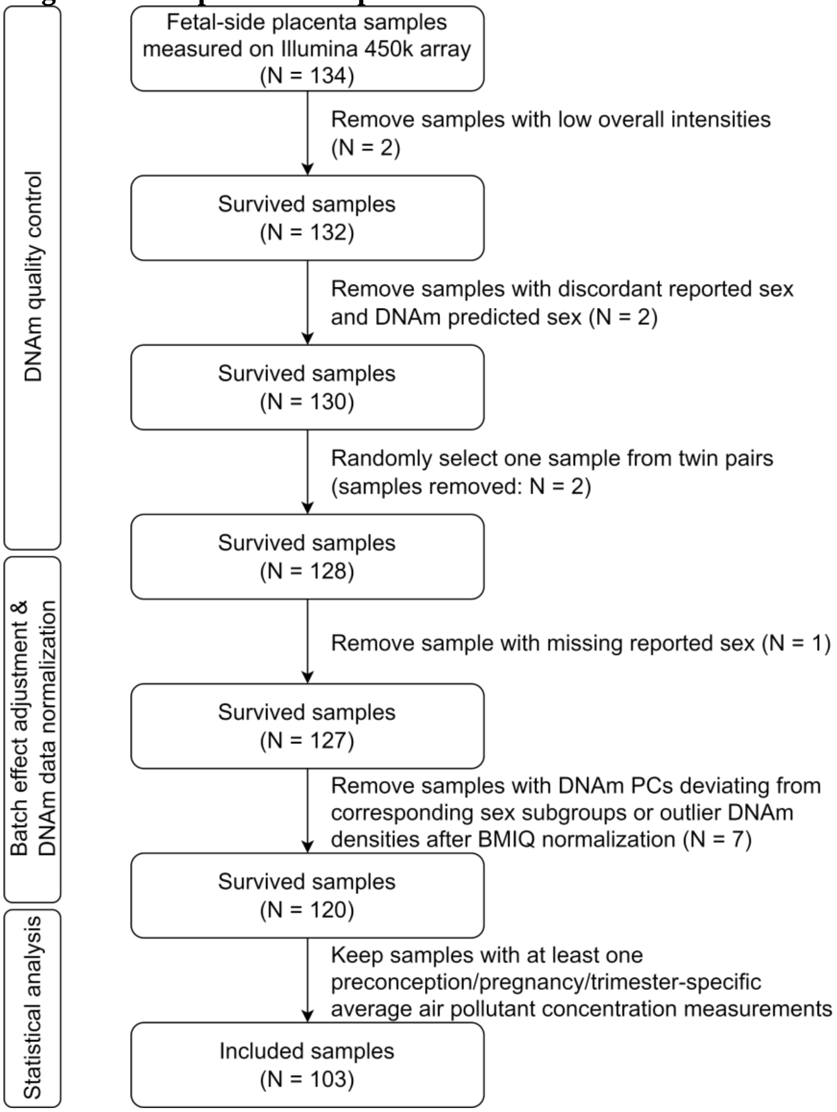

Abbreviations: DNAm, DNA methylation; PCs, principal components; BMIQ, Horvath's modified beta mixture quantile dilation method.

Figure S2. Conceptual framework on the associations of prenatal ambient air pollution with placental epigenetic gestational age at birth.

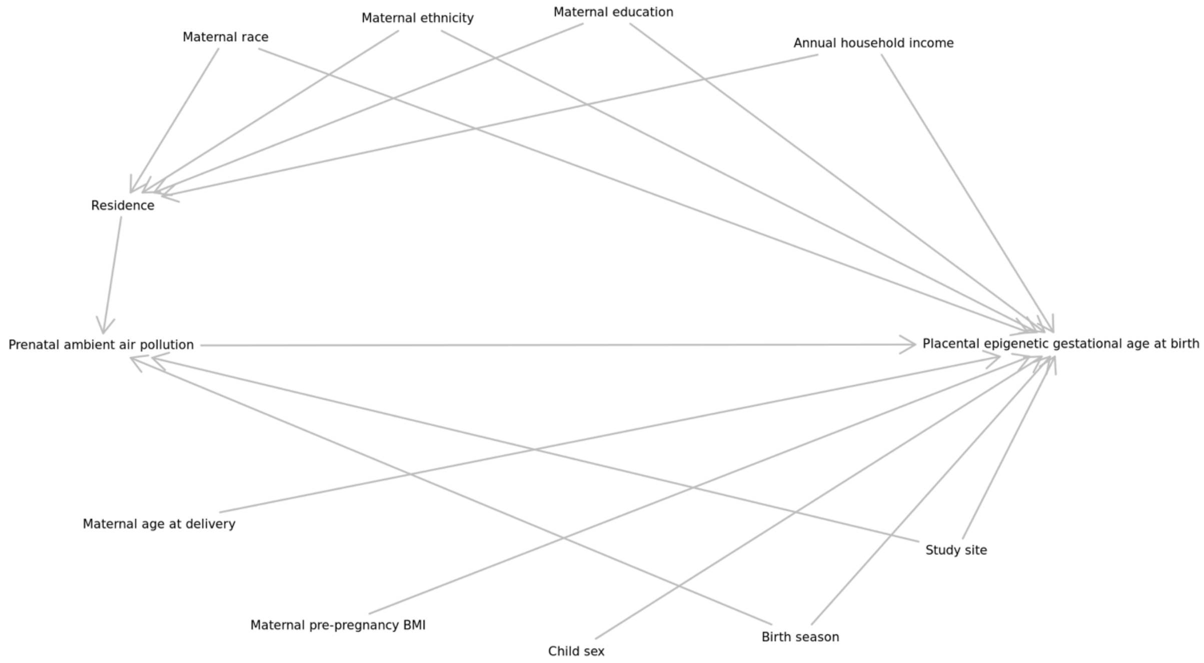

Abbreviations: BMI, body mass index.

Figure S3. Box and whisker plots of ambient air pollutant levels across exposure periods.

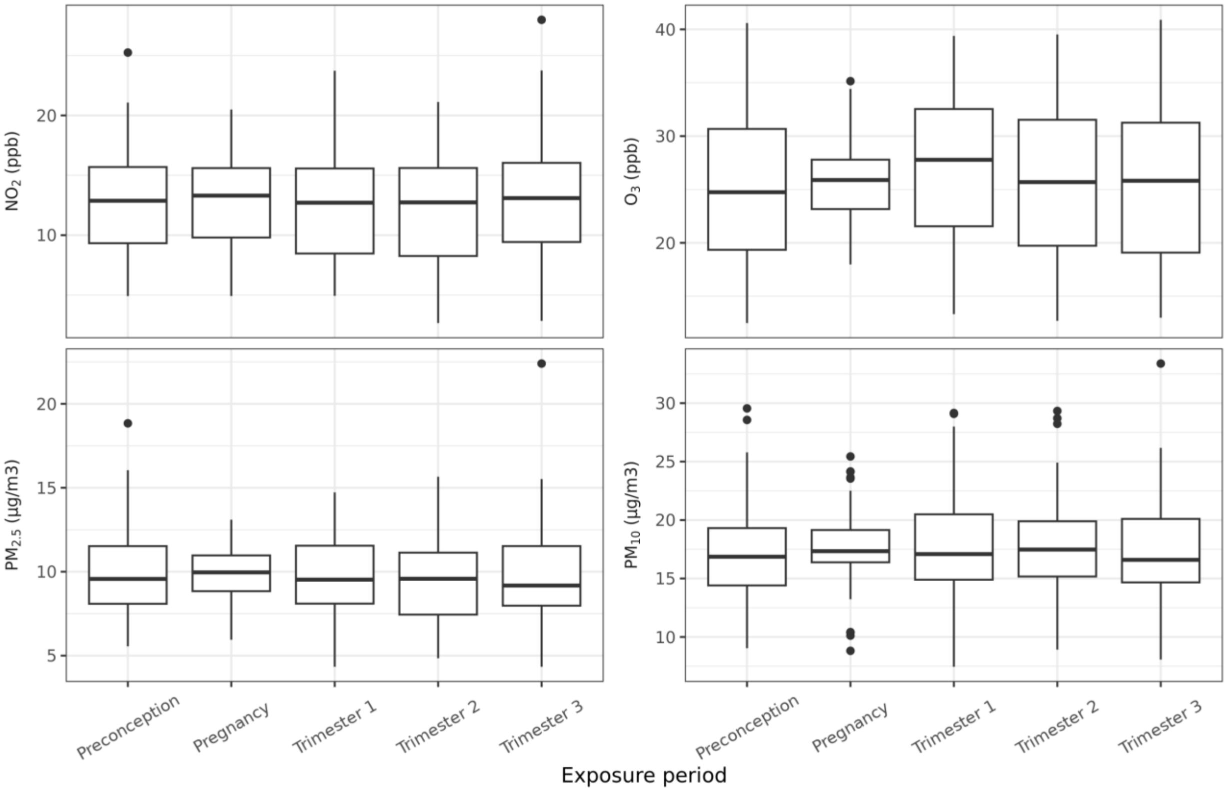

Abbreviations: NO_2, nitrogen dioxide; O_3, ozone; $PM_{2.5}$, particulate matter less than 2.5 microns in diameter; PM_{10}, particulate matter less than 10 microns in diameter; ppb, parts per billion; μg/m^3, microgram per cubic meter of air.

Boxes represent the interquartile ranges for air pollutants in each exposure period. Horizontal black lines in each box represent the median.

Figure S4. Box and Whisker plots of ambient air pollutant levels stratified by coastal regions.

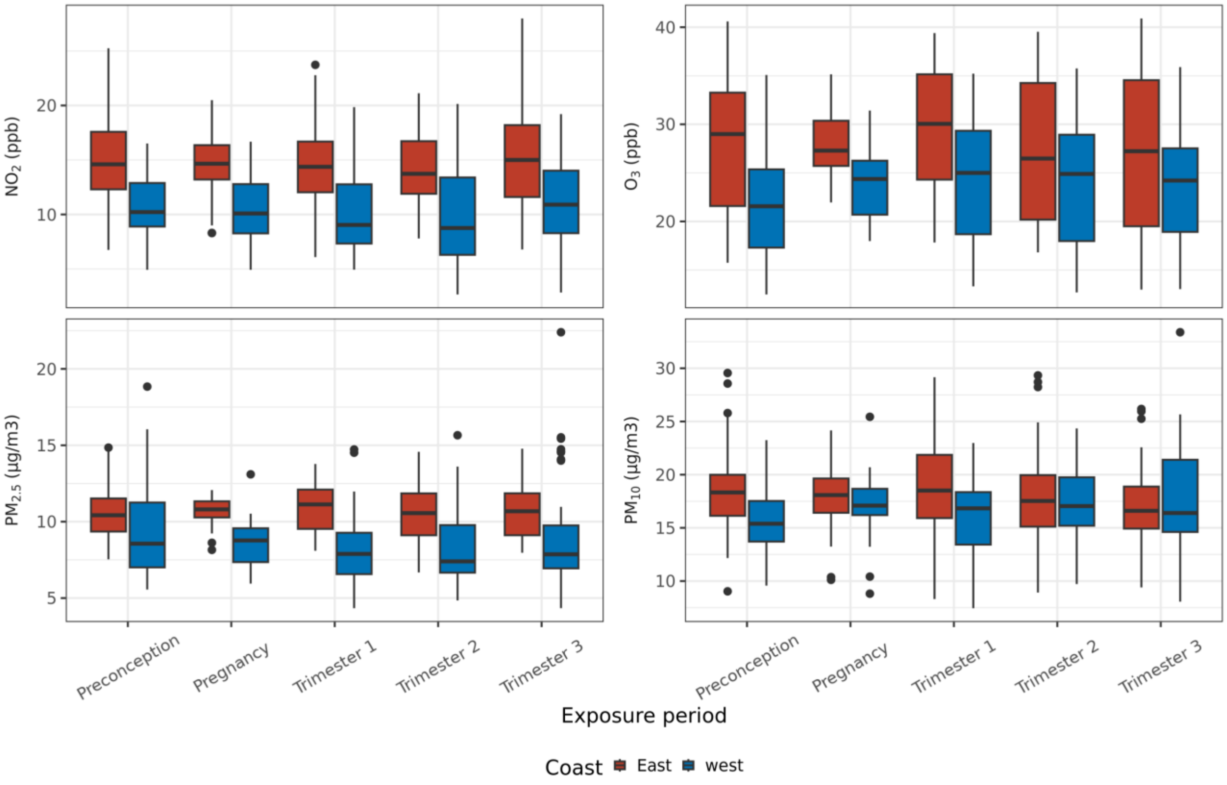

Abbreviations: NO_2, nitrogen dioxide; O_3, ozone; $PM_{2.5}$, particulate matter less than 2.5 microns in diameter; PM_{10}, particulate matter less than 10 microns in diameter; ppb, parts per billion; µg/m^3, microgram per cubic meter of air.

Boxes represent the interquartile ranges for air pollutants in each exposure period. Horizontal black lines in each box represent the median.

Figure S5. Correlations of average air pollutant levels within each exposure period.

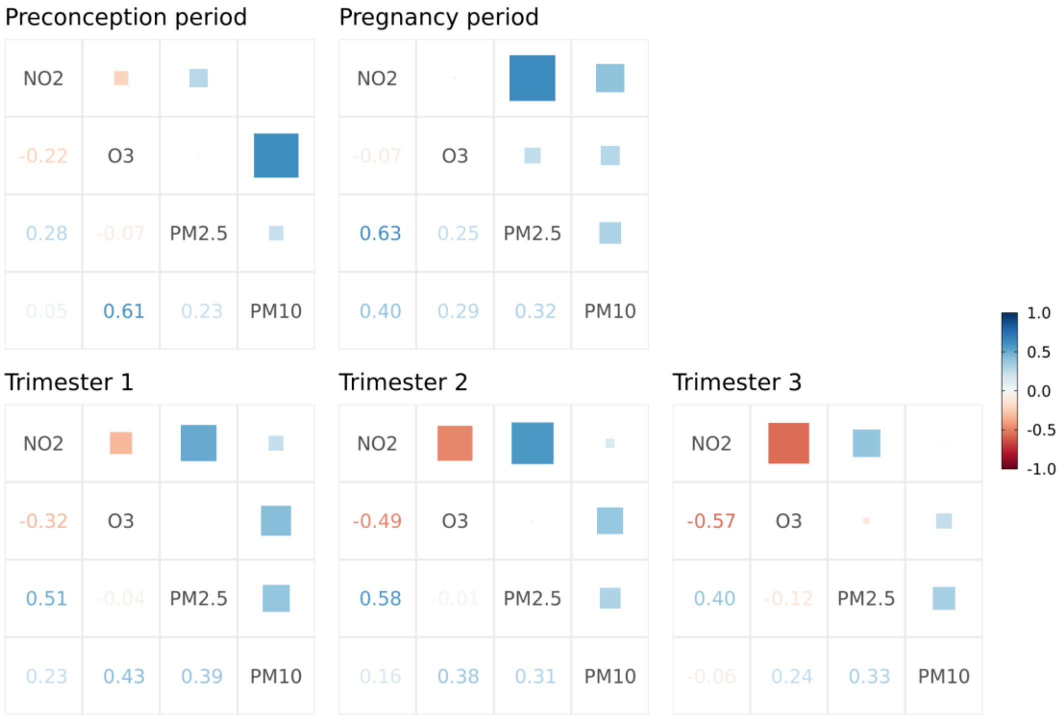

Abbreviations: NO_2, nitrogen dioxide; O_3, ozone; $PM_{2.5}$, particulate matter less than 2.5 microns in diameter; PM_{10}, particulate matter less than 10 microns in diameter.

Larger and darker shades represent stronger correlations. Blue shades indicate positive correlations, while red shades indicate negative correlations.

Figure S6. Correlations of average levels of each air pollutant across exposure periods.

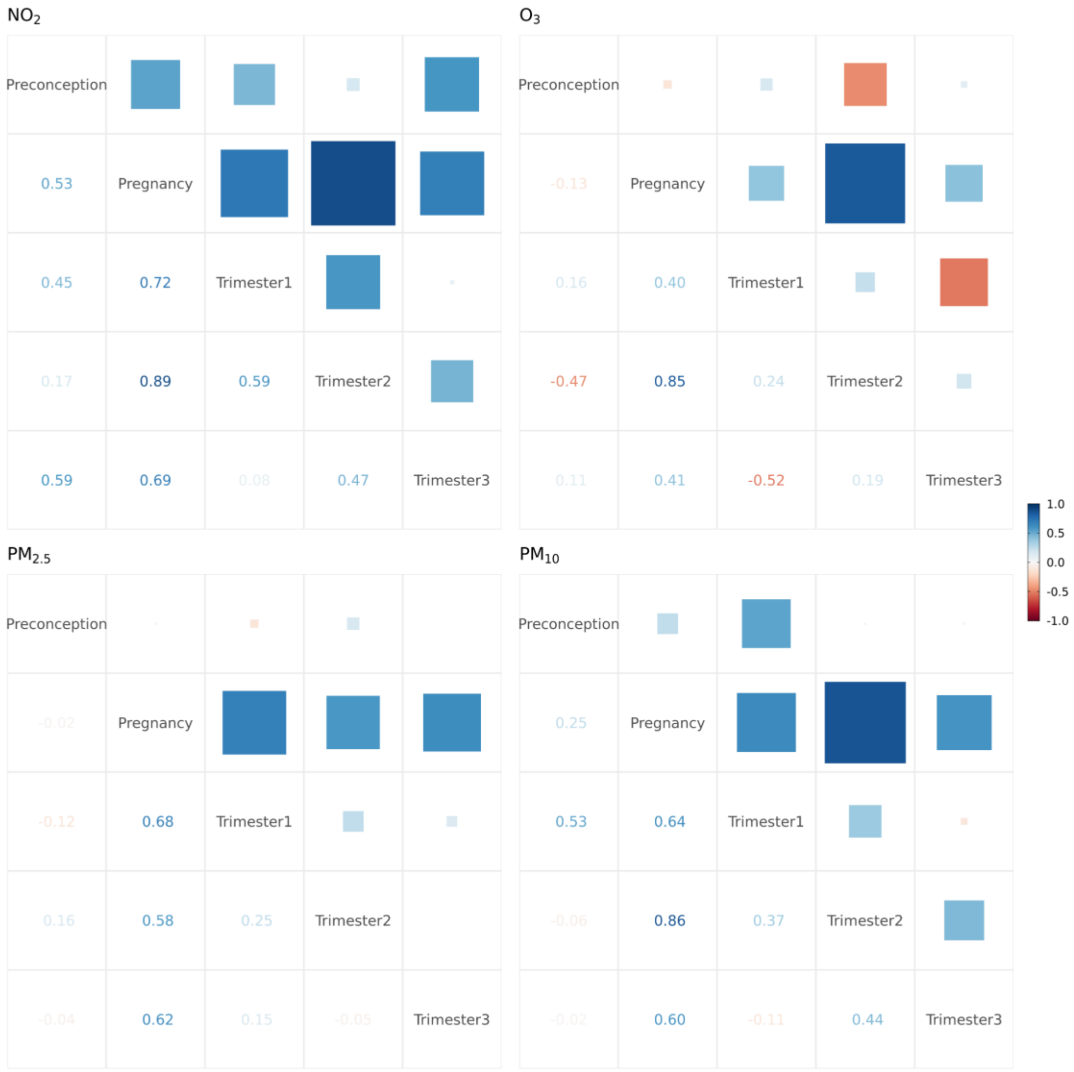

Abbreviations: NO_2, nitrogen dioxide; O_3, ozone; $PM_{2.5}$, particulate matter less than 2.5 microns in diameter; PM_{10}, particulate matter less than 10 microns in diameter.

Larger and darker shades represent stronger correlations. Blue shades indicate positive correlations, while red shades indicate negative correlations.

Figure S7. Correlations between chronologic gestational age and epigenetic gestational age.

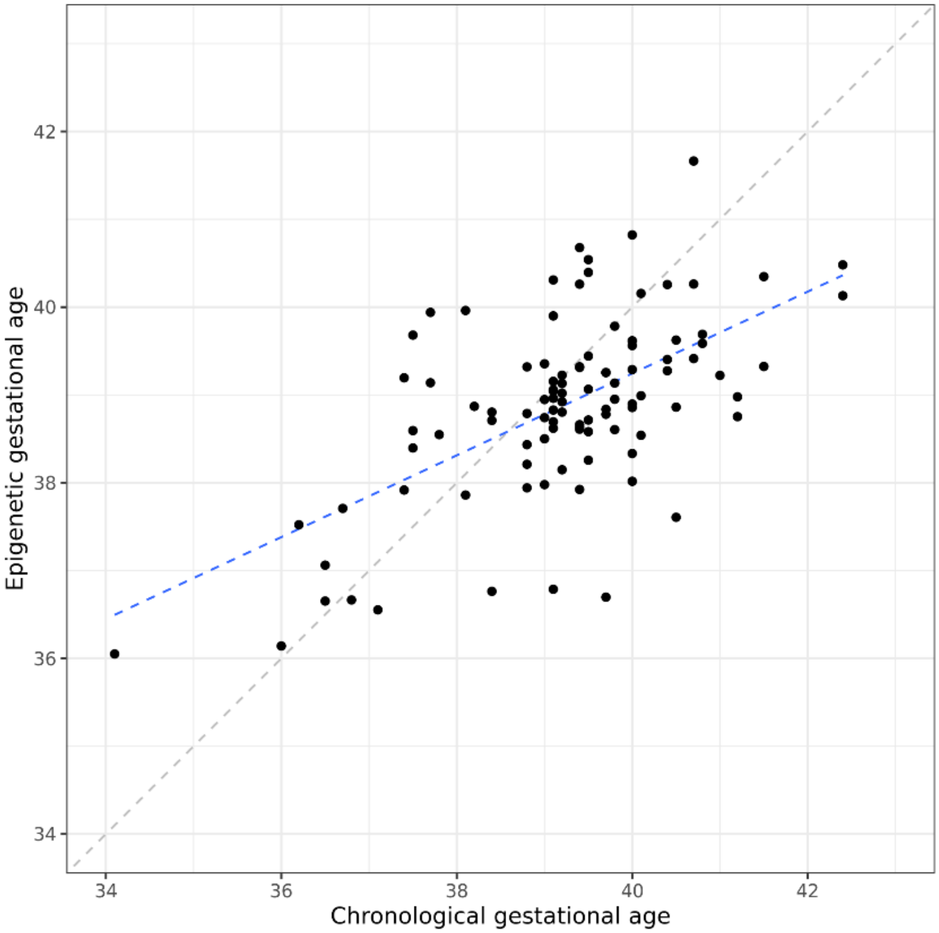

Scatter plots show the correlations between chronologic and epigenetic gestational age. Dashed blue lines in each panel are fitted univariate linear regression models with variable on the y-axis as dependent variable and variable on the x-axis as independent variable.

Figure S8. Adjusted associations for prenatal weekly ambient air pollution and placental epigenetic gestational age at birth, with polynomial distributed lag models for (A) NO_2 (n samples = 84) and (B) $PM_{2.5}$ (n samples = 87).

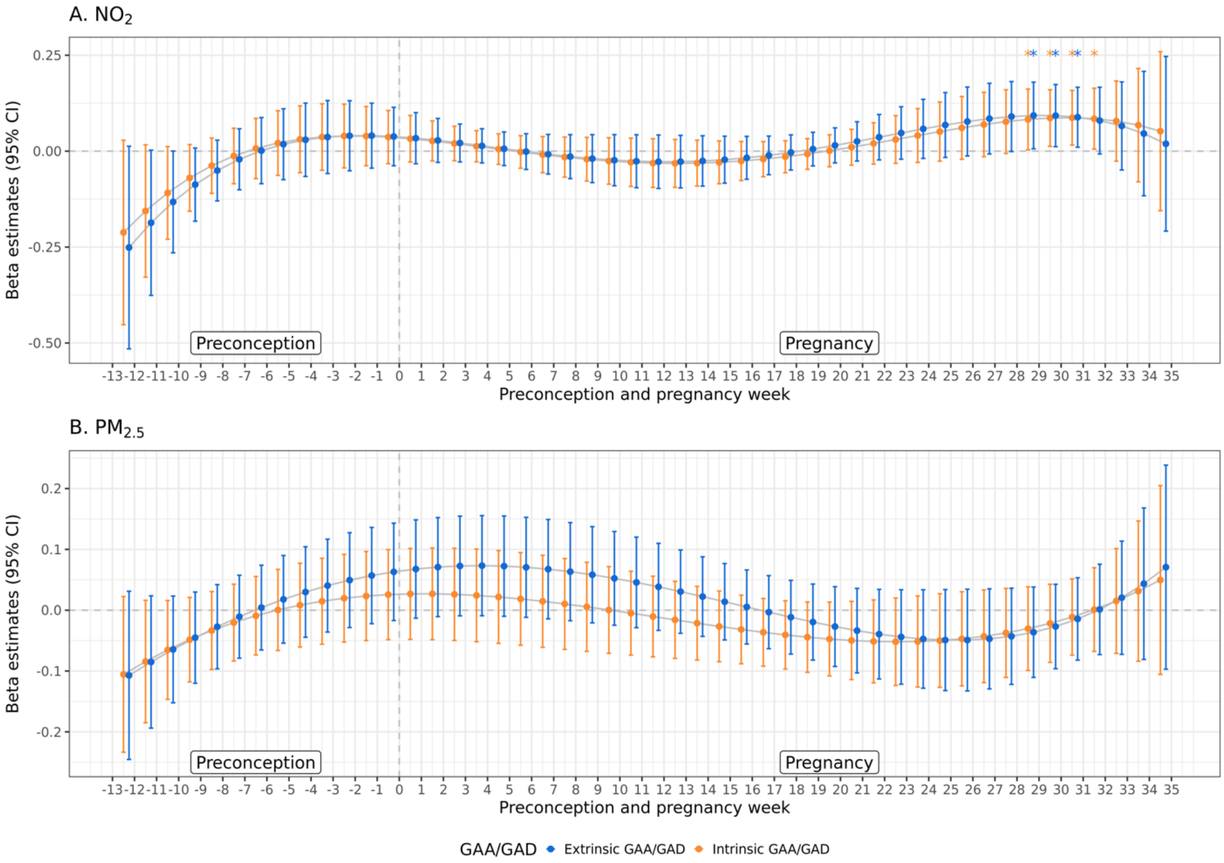

Abbreviations: NO_2, nitrogen dioxide; $PM_{2.5}$, particulate matter less than 2.5 microns in diameter; CI, confidence interval.

Figure S9. Adjusted associations for prenatal weekly ambient air pollution and placental epigenetic gestational age at birth, with natural cubic spline distributed lag models for (A) O_3 (n samples = 82) and (B) PM_{10} (n samples = 85).

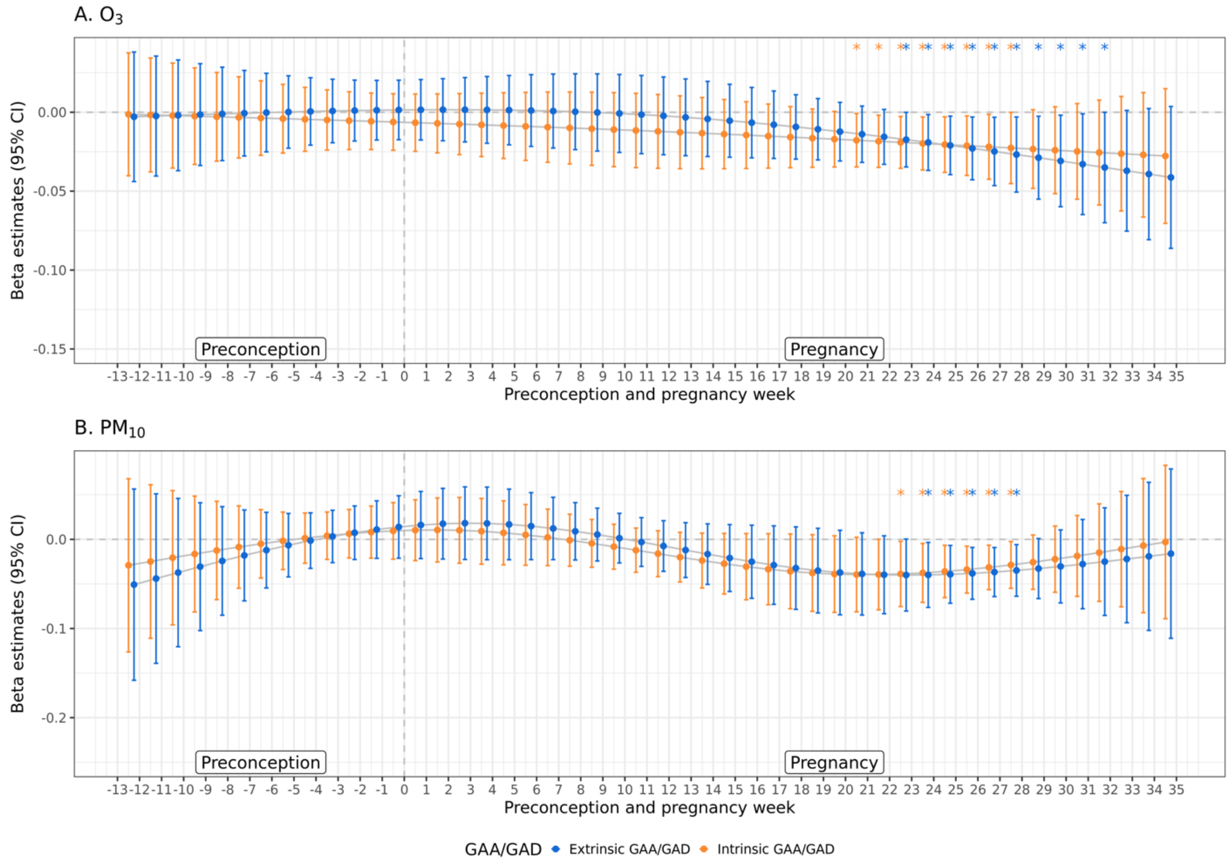

Abbreviations: O_3, ozone; PM_{10}, particulate matter less than 10 microns in diameter; CI, confidence interval.

Figure S10. Adjusted associations for prenatal weekly ambient air pollution and placental epigenetic gestational age at birth, with natural cubic spline distributed lag models for (A) NO_2 (n samples = 84) and (B) $PM_{2.5}$ (n samples = 87).

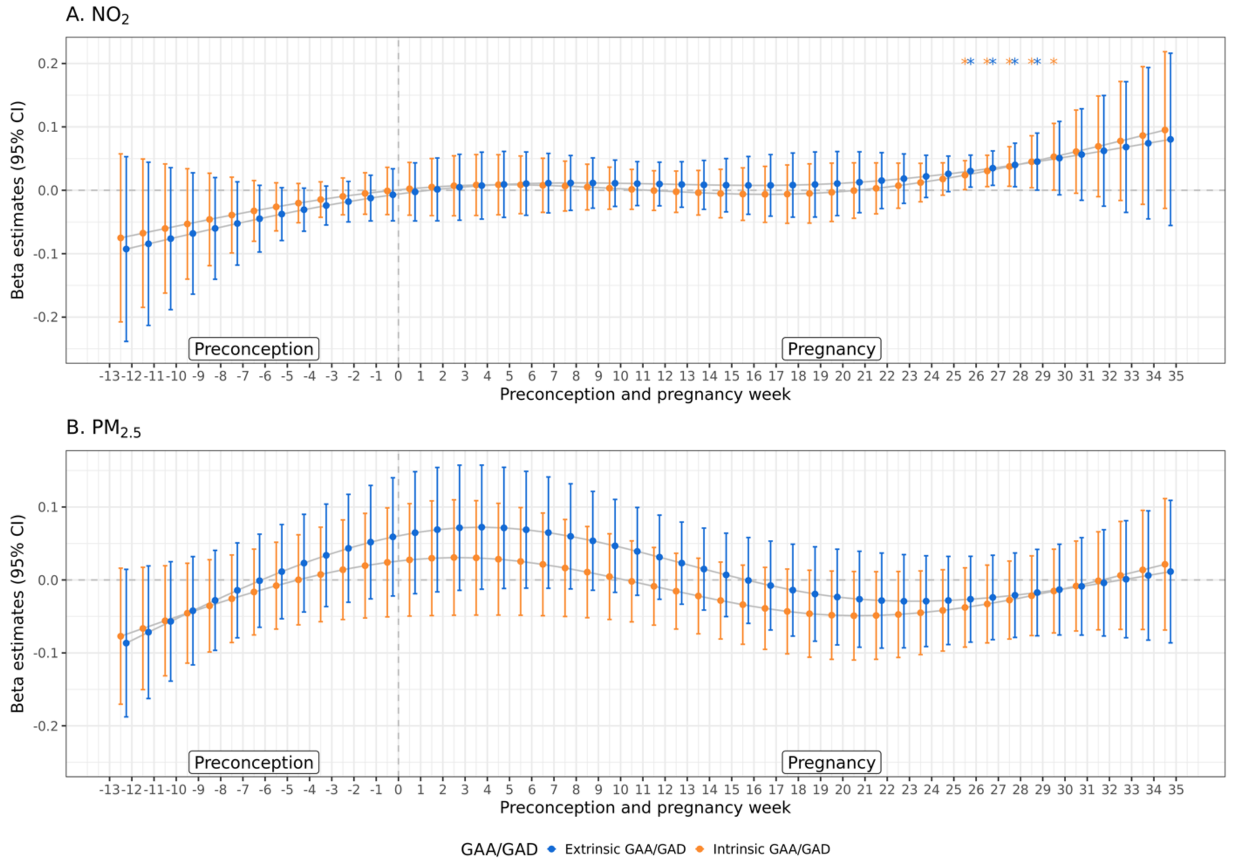

Abbreviations: NO_2, nitrogen dioxide; $PM_{2.5}$, particulate matter less than 2.5 microns in diameter; CI, confidence interval.

Figure S11. Adjusted associations for prenatal weekly ambient air pollution and placental epigenetic gestational age at birth among children born at full term, with polynomial distributed lag models for (A) O_3 (n samples=76) and (B) PM_{10} (n samples=79).

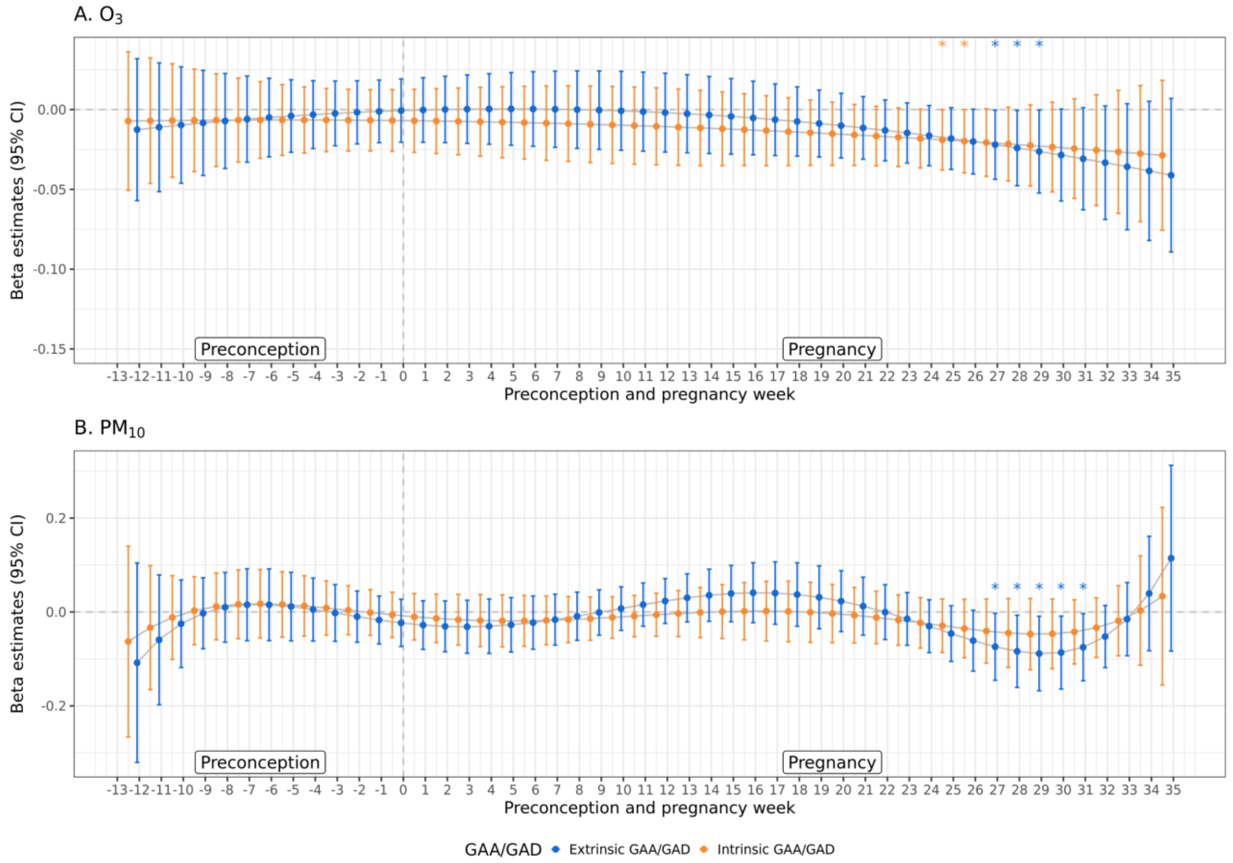

Abbreviations: O3, ozone in parts per billion (ppb); PM10, particulate matter less than 10 microns in diameter in micrograms per cubic meter of air (μg/m^3); CI, confidence interval.

Figure S12. Adjusted associations for prenatal weekly ambient air pollution and placental epigenetic gestational age at birth among children born at full term, with polynomial distributed lag models for (A) NO_2 (n samples = 78) and (B) $PM_{2.5}$ (n samples = 81).

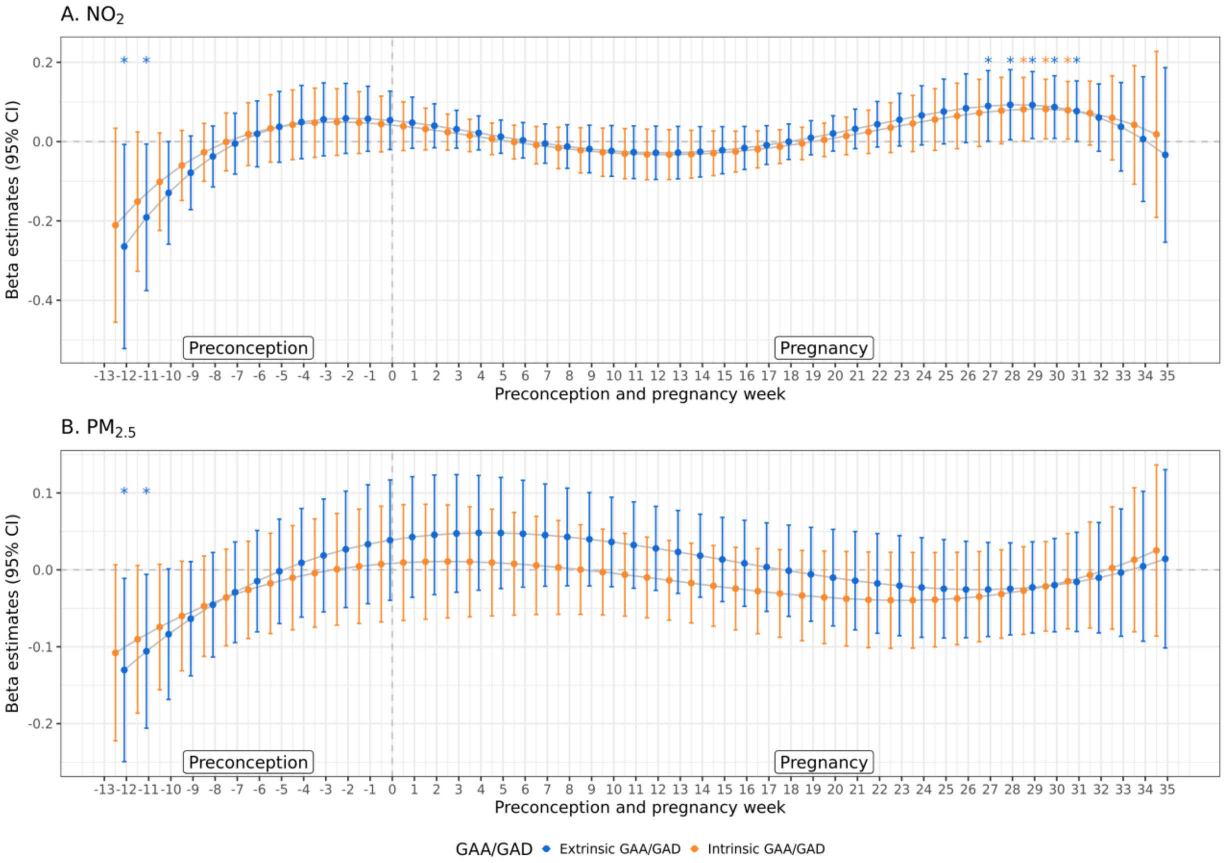

Abbreviations: NO_2, nitrogen dioxide; $PM_{2.5}$, particulate matter less than 2.5 microns in diameter; CI, confidence interval.

Figure S13. Adjusted associations for prenatal weekly ambient air pollution and placental epigenetic gestational age at birth among children born at full term, with natural cubic spline distributed lag models for (A) O_3 (n samples = 76) and (B) PM_{10} (n samples = 79).

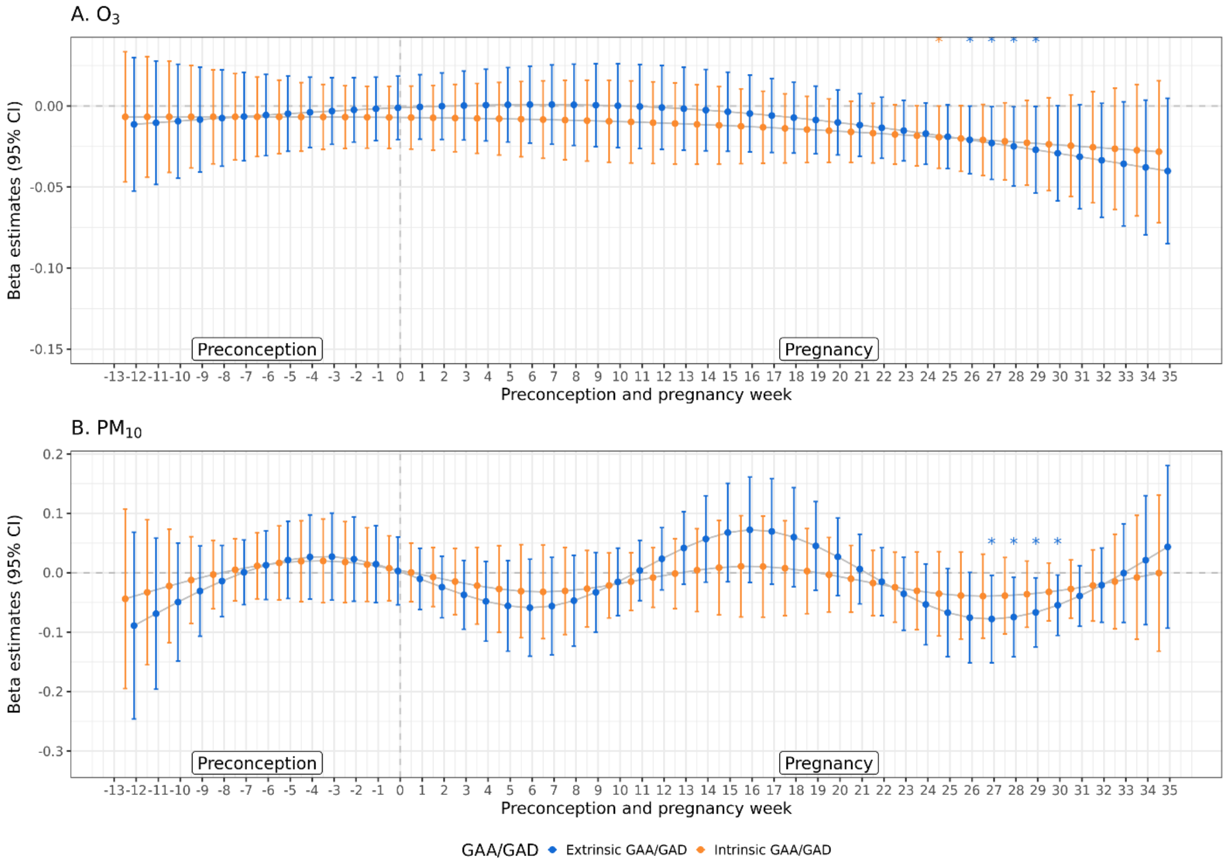

Abbreviations: O_3, ozone; PM_{10}, particulate matter less than 10 microns in diameter; CI, confidence interval.

Figure S14. Adjusted associations for prenatal weekly ambient air pollution and placental epigenetic gestational age at birth among children born at full term, with natural cubic spline distributed lag models for (A) NO_2 (n samples = 78) and (B) $PM_{2.5}$ (n samples = 81).

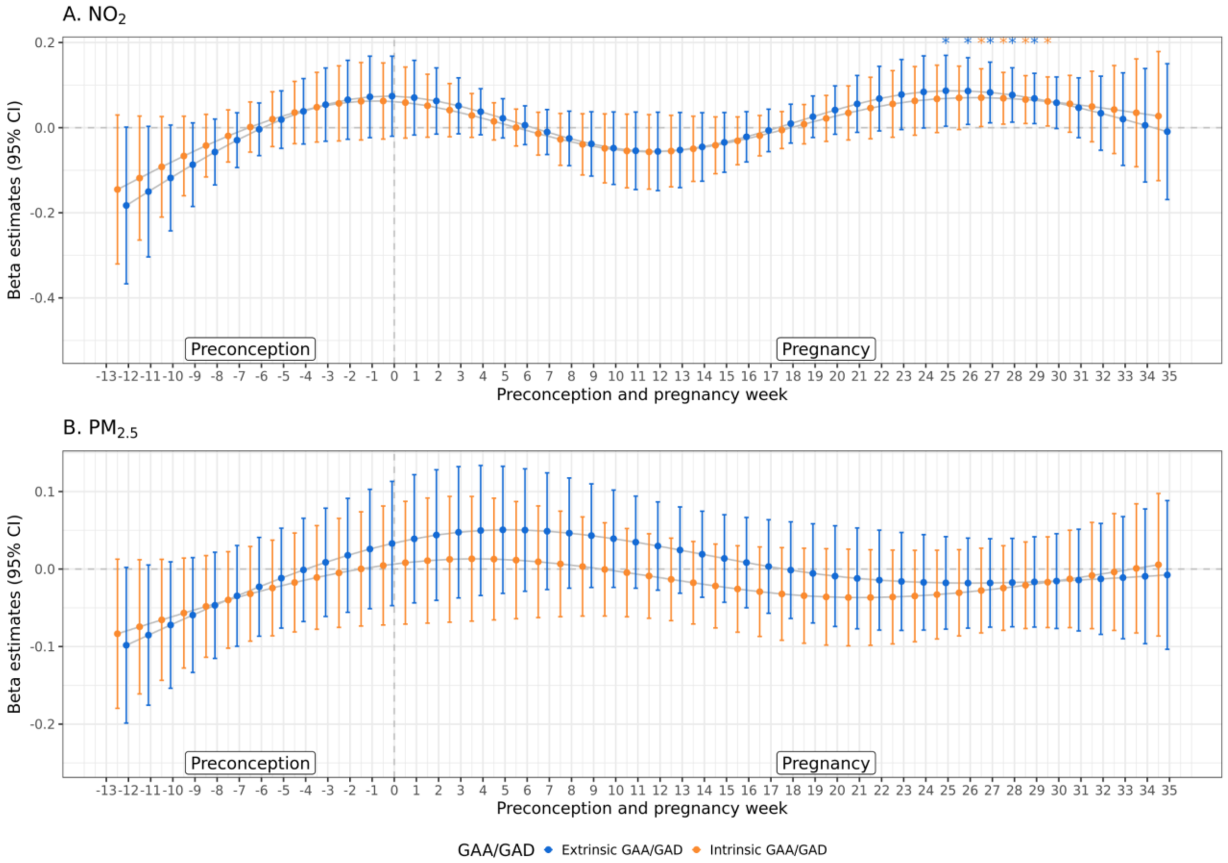

Abbreviations: NO_2, nitrogen dioxide; $PM_{2.5}$, particulate matter less than 2.5 microns in diameter; CI, confidence interval.
